# Neil3-dependent base excision repair regulates lipid metabolism and prevents atherosclerosis in Apoe-deficient mice

**DOI:** 10.1038/srep28337

**Published:** 2016-06-22

**Authors:** Tonje Skarpengland, Sverre Holm, Katja Scheffler, Ida Gregersen, Tuva B. Dahl, Rajikala Suganthan, Filip M. Segers, Ingunn Østlie, Jeroen J. T. Otten, Luisa Luna, Daniel F. J. Ketelhuth, Anna M. Lundberg, Christine G. Neurauter, Gunn Hildrestrand, Mona Skjelland, Bodil Bjørndal, Asbjørn M. Svardal, Per O. Iversen, Ulf Hedin, Ståle Nygård, Ole K. Olstad, Kirsten Krohg-Sørensen, Geir Slupphaug, Lars Eide, Anna Kuśnierczyk, Lasse Folkersen, Thor Ueland, Rolf K. Berge, Göran K. Hansson, Erik A. L. Biessen, Bente Halvorsen, Magnar Bjørås, Pål Aukrust

**Affiliations:** 1 Research Institute of Internal Medicine, Oslo University Hospital Rikshospitalet, Oslo, Norway; 2 Institute of Clinical Medicine, University of Oslo, Oslo, Norway; 3 Department of Medical Biochemistry, Oslo University Hospital Rikshospitalet, Oslo, Norway; 4 Institute of Basic Medical Research, University of Oslo, Oslo, Norway; 5 Department of Informatics, University of Oslo, Oslo, Norway; 6 K.G. Jebsen Inflammatory Research Center, University of Oslo, Oslo, Norway; 7 Department of Microbiology, Oslo University Hospital Rikshospitalet, Oslo, Norway; 8 Department of Pathology,Oslo University Hospital Radiumhospitalet, Oslo, Norway; 9 Department of Experimental Vascular Pathology, University of Maastricht, Maastricht, The Netherlands; 10 Department of Surgery, Karolinska University Hospital, Stockholm, Sweden; 11 Center for Molecular Medicine, Karolinska University Hospital, Stockholm, Sweden; 12 Department of Neurology, Oslo University Hospital Rikshospitalet, Oslo, Norway; 13 Department of Clinical Science, University of Bergen, Bergen, Norway; 14 Department of Hematology, Oslo University Hospital Rikshospitalet, Oslo, Norway; 15 Department of Nutrition, University of Oslo, Oslo, Norway; 16 Department of Medical Biochemistry, Oslo University Hospital Ullevål, Oslo, Norway; 17 Department of Thoracic and Cardiovascular Surgery, Oslo University Hospital Rikshospitalet, Oslo, Norway; 18 Department of Cancer Research and Molecular Medicine, Norwegian University of Science and Technology, Trondheim, Norway; 19 PROMEC Core Facility for Proteomics and Metabolomics, Norwegian University of Science and Technology, Trondheim, Norway; 20 Center for Biological Sequence Analysis, Technical University of Denmark, Copenhagen, Denmark; 21 Department of Heart Disease, Haukeland University Hospital, Bergen, Norway; 22 Section of Clinical Immunology and Infectious Diseases, Oslo University Hospital Rikshospitalet, Oslo, Norway

## Abstract

Increasing evidence suggests that oxidative DNA damage accumulates in atherosclerosis. Recently, we showed that a genetic variant in the human DNA repair enzyme *NEIL3* was associated with increased risk of myocardial infarction. Here, we explored the role of Neil3/NEIL3 in atherogenesis by both clinical and experimental approaches. Human carotid plaques revealed increased *NEIL3* mRNA expression which significantly correlated with mRNA levels of the macrophage marker *CD68. Apoe*^*−/−*^*Neil3*^*−/−*^ mice on high-fat diet showed accelerated plaque formation as compared to *Apoe*^*−/−*^ mice, reflecting an atherogenic lipid profile, increased hepatic triglyceride levels and attenuated macrophage cholesterol efflux capacity. *Apoe*^*−/−*^*Neil3*^*−/−*^ mice showed marked alterations in several pathways affecting hepatic lipid metabolism, but no genotypic alterations in genome integrity or genome-wide accumulation of oxidative DNA damage. These results suggest a novel role for the DNA glycosylase Neil3 in atherogenesis in balancing lipid metabolism and macrophage function, potentially independently of genome-wide canonical base excision repair of oxidative DNA damage.

Complications of atherosclerosis, including stroke and myocardial infarction (MI), are major causes of mortality and morbidity worldwide. Atherosclerosis is acknowledged as a multifactorial disease comprising a broad spectrum of interacting inflammatory and metabolic pathways^1^. However, all aspects of the underlying pathogenesis are far from clear and there is a need for delineating novel pathogenic mechanisms that could represent new targets for therapy in atherosclerotic disorders.

Over the past decades, oxidative stress has been widely accepted as an important feature of atherosclerosis. Oxidative stress induces both nuclear and mitochondrial DNA damage, and accumulation of DNA damage has been reported in atherosclerotic lesions^2^. If not counteracted, DNA damage may challenge the genomic integrity, leading to cellular dysfunction, senescence, mutagenesis or death^3^,^4^. Thus, DNA repair mechanisms represent molecular processes with a potential to influence the pathogenesis of atherosclerosis.

Base excision repair (BER) is the major pathway for removal of oxidized DNA bases^5^. BER is initiated by a lesion-specific DNA glycosylase that removes the damaged base to generate an abasic site (AP site). The potentially harmful AP site is further processed by various enzymatic activities, which either includes a nuclease that removes the base-less sugar-phosphate residue, or a flap endonuclease/helicase for excision of larger DNA fragments. BER is subsequently completed by incorporation of a native base by a polymerase, and finally a ligase-mediated sealing of the DNA strand^5^. At least five different DNA glycosylases, including NEIL1, NEIL2 and NEIL3, with partly overlapping substrate affinities are involved in the removal of oxidized DNA bases. Moreover, oxidative base lesion-specific glycosylases in mammals are bifunctional and have intrinsic lyase activity, thus generating strand breaks rather than abasic sites. Whereas the majority of these glycosylases are ubiquitously expressed, murine *Neil3* expression is age-dependent and restricted to discrete regions harboring proliferating cells, including bone marrow, spleen and cerebral niches of stem cell populations^6^,^7^,^8^. Neil3 also appears to play a role in regeneration after cerebral hypoxic ischemic injury^9^.

Studies exploring the role of DNA repair, and BER in particular, in atherosclerosis are limited. Based on NEIL3′s role in repair of oxidative DNA damage and its impact on cell proliferation and regeneration following ischemic injury, we hypothesized that NEIL3 could play a role in diseases characterized by metabolic and oxidative stress such as atherosclerosis. Here, this hypothesis was investigated by several experimental approaches, including examination of samples from patients with carotid atherosclerosis, studies of Neil3 deficiency in atherosclerosis-prone mice and *in vitro* experiments in relevant cell lines.

## Results

### Increased *NEIL3* expression in human carotid plaques

**Figure 1:**
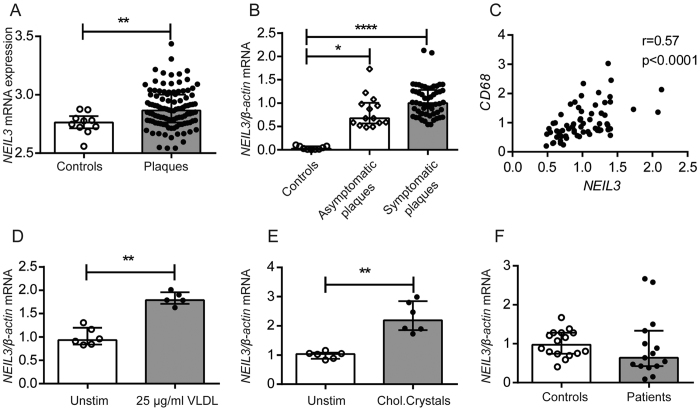
Regulation of *NEIL3* in clinical carotid atherosclerosis. (**A**) Relative *NEIL3* expression in human control arteries (n = 10) and carotid atherosclerotic plaques (n = 106) from the Biobank of Karolinska Endarterectomies (BiKE), as evaluated by microarray. (**B**) Relative *NEIL3* expression in human control arteries (n = 9) as well as in asymptomatic (n = 14) and symptomatic (n = 54) carotid plaques from the Biobank of Oslo, as evaluated by RT-qPCR. (**C**) The correlation between mRNA levels of *NEIL3* and *CD68* in carotid plaques (n = 67, Biobank of Oslo). (**D**) *NEIL3* expression in THP-1 macrophages that were exposed to VLDL (25 μg/ml) and (**E**) cholesterol crystals (100 μg/ml) for 6 hours (n = 5–6). (**F**) Relative *NEIL3* expression in PBMC isolated from healthy controls (n = 16) and patients with ischemic stroke (n = 15). mRNA levels in (**B**–**F**) were quantified by RT-qPCR and normalized to *β-ACTIN*. Experiments in (**D**,**E**) were repeated twice with similar findings. Data are presented as single values, median and interquartile range. *p < 0.05, **p < 0.01, ***p < 0.001 and ****p < 0.0001.

We first examined if *NEIL3* gene expression was regulated in clinical atherosclerosis by assessing transcript levels in human carotid plaques of two different study populations (i.e., Biobank of Karolinska Endarterectomies [BiKE] of Stockholm [Supplementary Table S1]^10^ and Biobank of Oslo [Supplementary Table S2]). In BiKE, microarray analyses revealed increased *NEIL3* expression in plaques (n = 106) as compared to non-atherosclerotic control arteries (n = 10, iliac arteries from organ donors, 3 females/7 males, median age 54 years, range 30–61 years) (Fig. 1A). These findings were replicated by RT-qPCR analysis of carotid plaques (n = 68) and non-atherosclerotic control arteries (n = 9, iliac arteries from organ donors, 2 females/7 males, median age 37 years, range 15–62 years) of the Biobank of Oslo, with enhanced *NEIL3* mRNA levels in carotid plaques (Fig. 1B). There was no significant difference between symptomatic (i.e., unstable plaques, n = 54) and asymptomatic (i.e., stable plaque, n = 14) lesions, categorized according to presence or absence of relevant clinical symptoms within the last 6 months, suggesting that the regulation of NEIL3 in human carotid plaques is related to the chronic atherosclerotic process rather than to plaque destabilization (Fig. 1B). Whereas the controls and patients were comparable in relation to gender, the organ donors were younger. Of ethical reasons it is not possible to obtain control samples from carotid arteries, and samples collected during autopsy will be unreliable due to degradation of mRNA. Nonetheless, difference in age between patients and controls is a limitation of our analyses.

The plaques from the Biobank of Oslo were further analyzed and revealed a significant positive correlation between mRNA levels of *NEIL3* and the macrophage marker *CD68* (Fig. 1C). Further, THP-1 macrophages exposed to lipids, i.e., VLDL and cholesterol crystals showed increased *NEIL3* expression (Fig. 1D,E).

In contrast to the findings within the arterial specimens, PBMC obtained from 15 patients undergoing carotid endarterectomy (Biobank of Oslo) and from 16 healthy individuals showed similar *NEIL3* expression (Fig. 1F), suggesting that the increased expression of *NEIL3* in carotid plaques is regulated within the atherosclerotic lesion rather than in circulating mononuclear cells. Although the “PBMC patients” were recruited from the same cohort as the “plaque patients”, PBMC and plaques were obtained from different patients, weakening the impact of this finding.

### Increased atherosclerosis in male *Apoe* ^*−/−*^ *Neil3* ^*−/−*^ mice on high-fat diet

**Figure 2:**
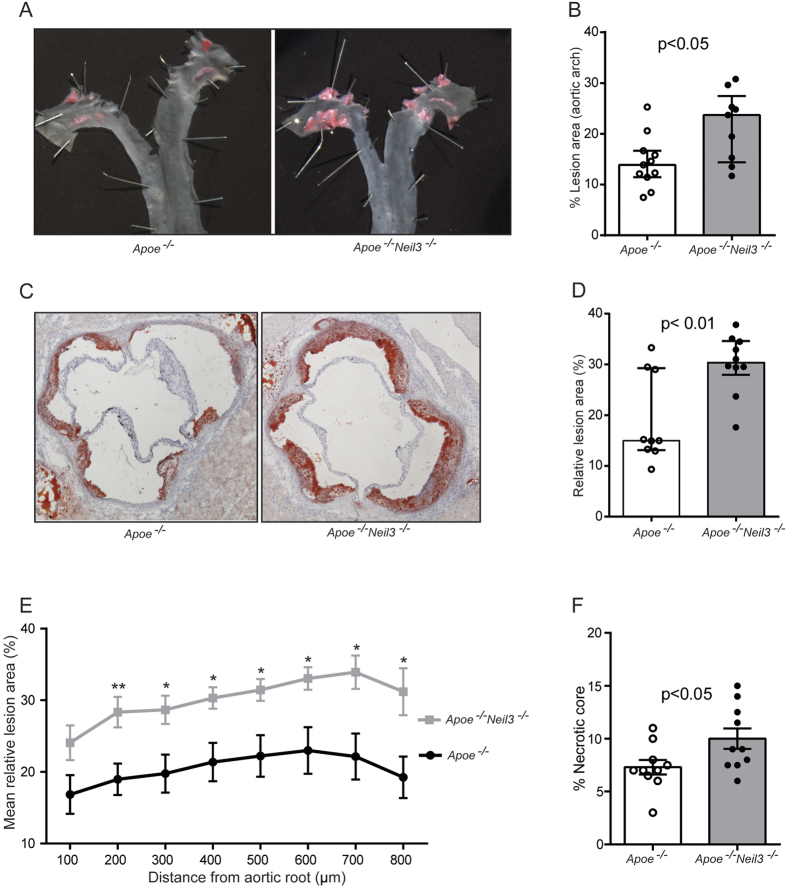
Neil3 deficiency on an *Apoe*^*−/−*^ background augments atherosclerosis in male mice fed a high-fat diet. (**A**) Representative *en face* images of the aortic arch stained with Sudan IV. (**B**) Data show *en face* % lesion area of *Apoe*^*−/−*^ (n = 11) and *Apoe*^*−/−*^*Neil3*^*−/−*^ (n = 9) mice. (**C**) Representative cryosections (10 μm) of the aortic root, stained with Oil Red O and hematoxylin. Original magnification 40X. (**D**) Relative lesion areas (lesion area/area inside external elastic lamina × 100) in cross-sections of the aortic root, calculated from 8 consecutive sections per mouse at 100 μm intervals in *Apoe*^*−/−*^ (n = 9) and *Apoe*^*−/−*^*Neil3*^*−/−*^ (n = 10) mice. (**E**) The graph shows the mean and SEM of relative lesion areas at 8 different positions in the aortic root; n = 9–11 (*Apoe*^*−/−*^) and n = 10 (*Apoe*^*−/−*^*Neil3*^*−/−*^), respectively. *p < 0.05 and **p < 0.01 versus *Apoe*^*−/−*^ mice. (**F**) Necrotic core area as percentage of total plaque area in *Apoe*^*−/−*^ (n = 10) and *Apoe*^*−/−*^*Neil3*^*−/−*^ (n = 10) mice. Data in (**B**,**D**,**F)** are presented as single values, median and interquartile range and were analyzed using Mann-Whitney U test.

To elucidate the potential role of enhanced *NEIL3* expression in human carotid plaques, we examined the effect of *Neil3* deficiency in experimental atherosclerosis. Male *Apoe*^*−/−*^*Neil3*^*−/−*^ and *Apoe*^*−/−*^ mice were fed high-fat diet for 18 weeks to accelerate plaque formation and metabolic and oxidative stress^11^,^12^,^13^,^14^,^15^. *En face* quantification of atherosclerosis in the aortic arch revealed significantly larger lesions in *Apoe*^*−/−*^*Neil3*^*−/−*^ as compared to *Apoe*^*−/−*^ mice (Fig. 2A,B). Congruent with the *en face* results, frozen sections of the aortic root displayed increased plaque formation in *Apoe*^*−/−*^*Neil3*^*−/−*^ mice (Fig. 2C–E), showing that Neil3 deficiency accelerates atherogenesis in male *Apoe*^*−/−*^ mice on high-fat diet.

Immunohistochemistry of the aortic lesions revealed significantly higher content of Cd68^+^ macrophages and Cd4^+^ T cells in plaques of *Apoe*^*−/−*^*Neil3*^*−/−*^ mice although not significant when adjusted for plaque sizes (Supplementary Figure S1A–F). However, more advanced plaques may show an increase in relative necrotic core area, as also seen in the *Apoe*^*−/−*^*Neil3*^*−/−*^ mice (Fig. 2F), with reduced relative macrophage content, suggesting that Neil3 deficiency may in fact enhance the content of lesional macrophages. No differences in Cd8^+^ T cells, Vcam1-positive endothelial cells or α-actin positive smooth muscle cells (SMCs) were detected between the two genotypes (Supplementary Figure S1G–O). Neither bright field microscopy nor polarized light microscopy, showed differences in relative collagen content between the two groups (Supplementary Figure S2). Also, evaluation of the lesions with polarized light microscopy demonstrated similar levels of thick (red) and thin (green) collagen fibers (Supplementary Figure S2).

### An atherogenic lipid profile in *Apoe* ^*−/−*^ *Neil3* ^*−/−*^ mice

Assessment of the cytokine profile in plasma displayed only few changes, with decreased levels of both pro- (i.e., IL-1β and G-CSF) and anti- (i.e., IL-5) atherogenic cytokines in *Apoe*^*−/−*^*Neil3*^*−/−*^ mice (Supplementary Table S3). However, the *Neil3*^*−/−*^*Apoe*^*−/−*^ mice showed a markedly disturbed lipid profile in plasma/serum after 18 weeks on high-fat diet as compared to *Apoe*^*−/−*^ mice, with higher levels of TG, total cholesterol, free cholesterol, LDL cholesterol, oxidized LDL (oxLDL) cholesterol and HDL cholesterol (Supplementary Figure S3A–F). Other metabolic parameters, such as body weight, relative fat and lean body mass, as well as food intake and glucose levels were similar between the two genotypes (Supplementary Table S4).

### Increased levels of TG and monounsaturated fatty acids (MUFA) in the liver of *Apoe* ^*−/−*^ *Neil3* ^*−/−*^ mice

**Figure 3:**
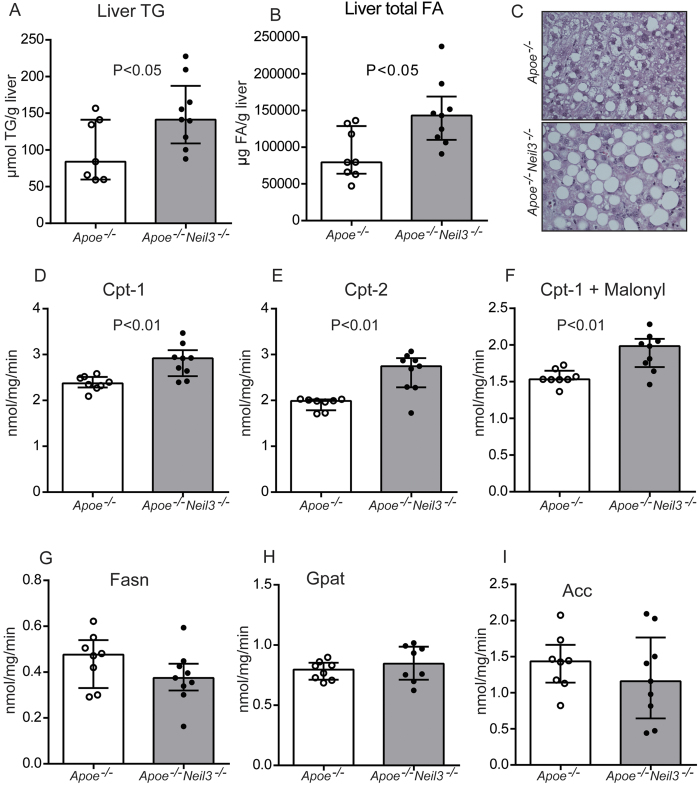
Increased hepatic lipids in *Apoe*^*−/−*^*Neil3*^*−/−*^ mice. Hepatic levels of (**A**) TG and (**B**) FA. (**C**) Representative images of liver histology of paraffin sections stained with hematoxylin and eosin. Panels (**D–I)** show the activity of hepatic enzymes involved in TG metabolism: Carnitine palmitoyltransferase-1 (Cpt-1), Cpt-2, Cpt-1 + malonyl, FA synthase (Fasn), glycerol-3-phosphate acyltransferase (Gpat) and acetyl-CoA carboxylase (Acc). Data are presented as single values, median and interquartile range.

The increased plasma levels of TG and cholesterol could potentially be related to altered hepatic lipid metabolism, and measurement of liver lipids in non-fasting mice revealed elevated TG and total FA levels in the *Apoe*^*−/−*^*Neil3*^*−/−*^ mice (Fig. 3A,B), without any significant changes in liver cholesterol and phospholipid levels (Supplementary Table S5). The accumulation of TG in *Apoe*^*−/−*^*Neil3*^*−/−*^ mice was accompanied by accelerated hepatic steatosis, as assessed by histological examination of liver specimens (Fig. 3C). The increased levels of FA in *Apoe*^*−/−*^*Neil3*^*−/−*^ mice reflected increased MUFA, mainly caused by increased levels of C18:1n-9 and C18:1n-7 (Supplementary Table S5). In contrast, the hepatic levels of total polyunsaturated FA (PUFA) were decreased in *Apoe*^*−/−*^*Neil3*^*−/−*^ mice, reflecting a significant reduction of n-9 PUFA and n-6 PUFA, including a decrease in C20:4n-6 (arachidonic acid) (Supplementary Table S5). Although n-3 PUFA levels were similar, C20:5n-3 (eicosapentaenoic acid), which has shown anti-atherogenic properties, was significantly reduced in *Apoe*^*−/−*^*Neil3*^*−/−*^ (Supplementary Table S5). Our findings suggest that the liver of *Apoe*^*−/−*^*Neil3*^*−/−*^ mice accumulates TG and FA, with an increase in MUFA and a decrease in PUFA, reflecting a pro-atherogenic lipid profile^16^.

**Figure 4:**
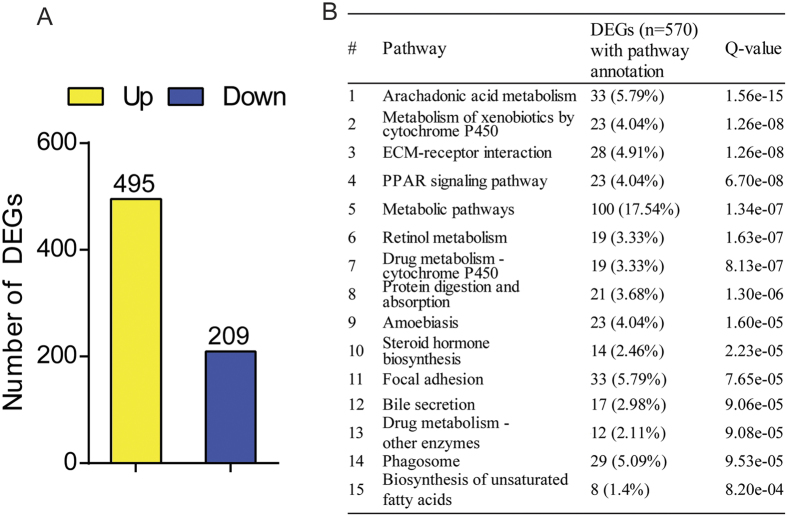
Neil3 deficiency modulates expression of hepatic genes. (**A**) The numbers of genes that were significantly up- and downregulated in *Apoe*^*−/−*^*Neil3*^*−/−*^ as compared to *Apoe*^*−/−*^ mice by RNA sequencing analysis. (**B**) KEGG pathway enrichment analyses showing the top 15 pathways significantly enriched in differentially expressed genes (DEGs). Percentages were calculated based on the total number of genes (n = 570) with pathway annotations. Q values are FDR-adjusted p-values and a Q value <0.05 was considered as significant. RNA sequencing was performed on pooled RNA from the liver of 7 *Apoe*^*−/−*^ and 9 *Apoe*^*−/−*^*Neil3*^*−/−*^ mice.

The increased TG levels could be caused by decreased hepatic FA processing via β-oxidation. However, the activities of the mitochondrial enzymes performing β-oxidation, i.e., carnitine palmitoyltransferase-1 (Cpt-1) and Cpt-2, were significantly enhanced in the *Apoe*^*−/−*^*Neil3*^*−/−*^ mice (Fig. 3D,E), and the Cpt-1 activity remained higher also in the presence of its natural key inhibitor malonyl-CoA (Fig. 4F). Altogether, these results suggest enhanced rather than decreased β-oxidation of long-chain FA in the *Apoe*^*−/−*^*Neil3*^*−/−*^ mice, but assessment of plasma carnitine metabolites revealed higher levels in the *Apoe*^*−/−*^*Neil3*^*−/−*^ mice of both palmitoyl-carnitine and the end product of β-oxidation, acetyl-carnitine (Supplementary Table S6), indicating that the increase in β-oxidation was unable to reduce the elevated TG levels. Moreover, the FA producing enzymes FA synthase (Fasn), glycerol-3-phosphate acyltransferase (Gpat) and acetyl-CoA carboxylase (Acc) all showed similar activities in both genotypes (Fig. 3G–I). Since there were no genotypic differences in relative body fat distribution or plasma free FA (Supplementary Table S4 and Table S6, respectively), the differences in TG levels were seemingly not induced by augmented FA release from peripheral fat tissues. In summary, elevated hepatic TG levels in *Apoe*^*−/−*^*Neil3*^*−/−*^ mice appeared not to be caused by decreased FA β-oxidation, augmented FA release from peripheral fat tissues or increased hepatic FA synthesis. Since hepatic MUFA and total FA levels were elevated, our data suggest that the increase in TG reflect enhanced substrate availability for TG synthesis rather than altered enzyme activity.

### Marked alterations in hepatic pathways involved in lipid metabolism in *Apoe* ^*−/−*^ *Neil3* ^*−/−*^ mice

In order to assess the general impact of Neil3 deficiency on all genes involved in liver metabolism, mRNA sequencing analysis was executed. In total, 495 and 209 hepatic genes were upregulated and downregulated in *Apoe*^*−/−*^*Neil3*^*−/−*^ mice, respectively, suggesting that Neil3 deficiency has a significant impact on hepatic gene expression (Fig. 4A). KEGG pathway enrichment analysis revealed that among the top 15 pathways significantly enriched in differently expressed genes (DEGs), several were involved in lipid metabolism (i.e., the arachidonic acid pathways, metabolism of xenobiotics, PPAR-related pathways, metabolic pathways, steroid hormone biosynthesis pathways, pathways related to bile secretion and pathways related to synthesis of unsaturated FA) (Fig. 4B). In summary, the atherogenic plasma lipid profile in the *Apoe*^*−/−*^*Neil3*^*−/−*^ mice could be related to increased hepatic levels of TG, total FA and MUFA, which were congruent with the RNA sequencing data of marked changes in several pathways related to hepatic lipid metabolism of *Apoe*^*−/−*^*Neil3*^*−/−*^ mice.

### Increased lipid-induced proliferation in *Apoe* ^*−/−*^ *Neil3* ^*−/−*^ macrophages

RNA sequencing analysis revealed higher levels of aortic *Cd68* (log2 = 1.19, FDR = 2.49e-278) and immunohistochemistry showed increased absolute number of Cd68^+^ cells in the aortic root of *Apoe*^*−/−*^*Neil3*^*−/−*^ mice (Supplementary Figure S1). Gene enrichment analyses of DEGs from thoracic aorta as identified by RNA sequencing revealed a phenotype associated with abnormal lipid homeostasis, a transcriptional profile similar to myeloid progenitors that differentiate into macrophages, and a macrophage-enriched metabolic network in the *Apoe*^*−/−*^*Neil3*^*−/−*^ mice (Supplementary Table S7). These findings suggest that macrophage-induced dysregulation of lipid homeostasis within the lesion could be of importance for the more advanced plaque in the *Apoe*^*−/−*^*Neil3*^*−/−*^ mice. The accumulation of macrophages within the lesion could reflect enhanced proliferative or migratory properties of *Apoe*^*−/−*^*Neil3*^*−/−*^ macrophages during plaque development. To mimic the lipid challenge of plaque macrophages, bone marrow-derived macrophages (BMDM) from both genotypes were stimulated with VLDL and oxLDL. As evaluated by RT-qPCR, *Ki67*, as a marker of cell proliferation, in BMDM from *Apoe*^*−/−*^*Neil3*^*−/−*^ mice but not from *Apoe*^*−/−*^ mice, showed enhanced expression after VLDL and oxLDL stimulation as compared to unstimulated cells (Supplementary Figure S4A). In contrast, no differences in the number of recruited peritoneal macrophages were detected after induction of sterile peritonitis with thioglyollate (Supplementary Figure S4B).

We could not detect any differences between the two genotypes in bone marrow long-term hematopoietic stem cells and multipotent progenitor cells (Supplementary Figure S5) and immunophenotypic screening by flow cytometry of lymph nodes, spleen and blood did not reveal any genotypic differences in absolute cell counts or leukocyte subsets (Supplementary Table S8). These results indicate that the increased number of plaque macrophages in *Apoe*^*−/−*^*Neil3*^*−/−*^ mice may involve genotypic cell-specific differences in proliferation in response to modified lipids within the lesion, and not altered recruitment from hematopoietic tissues or altered chemotactic properties.

### Reduced cholesterol efflux capacity in *Apoe* ^*−/−*^ *Neil3* ^*−/−*^ mice

**Figure 5:**
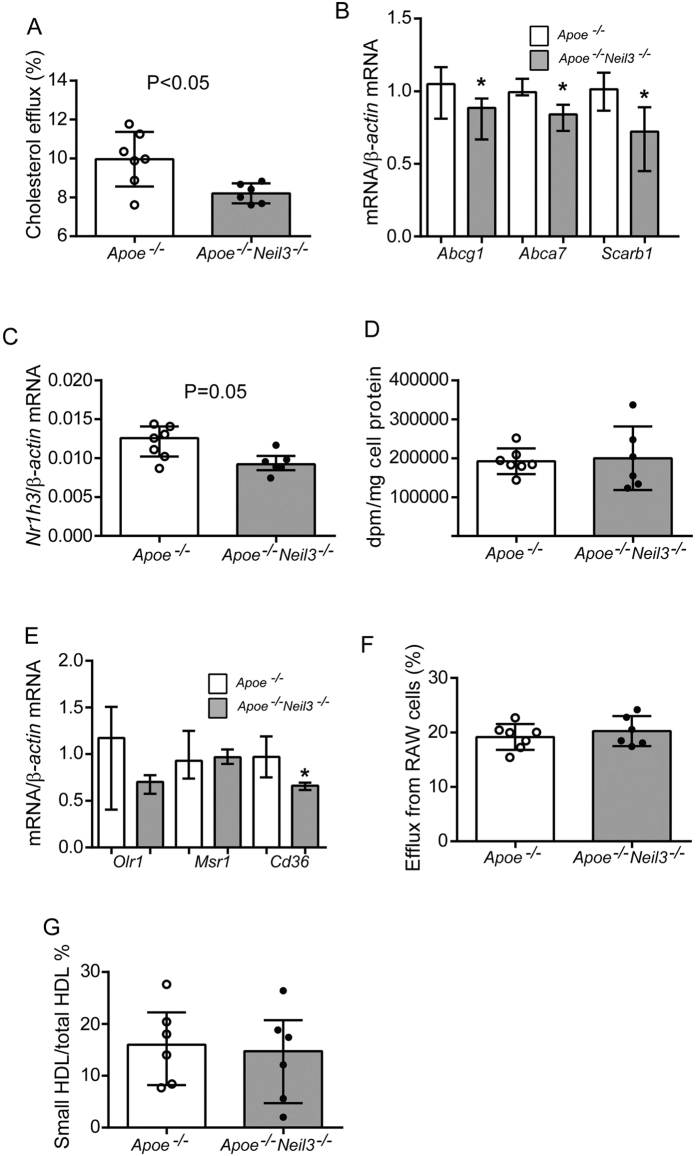
Decreased cholesterol efflux capacity in bone marrow-derived macrophages (BMDM) from *Apoe*^*−/−*^*Neil3*^*−/−*^ mice. (**A**) Cholesterol efflux capacity of BMDM from *Apoe*^*−/−*^ (n = 7) and *Apoe*^*−/−*^*Neil3*^*−/−*^ (n = 6) mice, using pooled serum from C57BL/6 wild type mice (n = 4) as cholesterol acceptor. The experiment was repeated twice with similar findings. (**B**,**C**) mRNA levels of genes involved in cholesterol efflux mechanisms; (n = 6-7 [*Abca7*, *Srb1* and *Nr1h3*] and n = 16–17 [*Abcg1*]). (**D**) BMBM were exposed to oxLDL (20 μg/ml) and ^14^C-cholesterol (0.5 μCi/ml) for 48 hours, washed with serum-free medium containing 0.2% BSA and the cells were lysed with 0.2 M NaOH. BMDM loading capacity was calculated as disintegrations per minute (dpm) per total protein. Values represent fractional (%) cholesterol efflux calculated as dpm (media)/dpm (media + cell-associated)] × 100. (**E**) mRNA levels of scavenger receptors in BMDM from *Apoe*^*−/−*^ (n = 6) and *Apoe*^*−/−*^*Neil3*^*−/−*^ (n = 3) mice. (**F**) Serum cholesterol acceptor capacity was evaluated by measuring cholesterol efflux from murine macrophages (RAW264.7 cells) using sera from *Apoe*^*−/−*^ (n = 7) and *Apoe*^*−/−*^*Neil3*^*−/−*^ (n = 6) mice as cholesterol acceptors. (**G**) The distribution of small plasma HDL cholesterol relative to all plasma HDL cholesterol. mRNA levels were quantified with RT-qPCR and normalized to β*-actin* as reference gene. Data are presented as single values, median and interquartile range. *p < 0.05.

In addition to lipid availability, the accumulation of lipid within the atherosclerotic lesion depends on lipid uptake and efflux capacity of lesional macrophages^17^. The cholesterol efflux capacity of BMDM of *Apoe*^*−/−*^*Neil3*^*−/−*^ mice was significantly decreased as compared to *Apoe*^*−/−*^ BMDM, using serum from pooled C57BL/6 wild type mice as cholesterol acceptor (Fig. 5A). Transcriptional assessment of the major actors of lipid efflux revealed decreased expression of *Abcg1, Abca7* and *Scarb1* (i.e., the murine ortholog of SR-B1) in BMDM from *Apoe*^*−/−*^*Neil3*^*−/−*^ mice (Fig. 5B). Finally, the murine ortholog of LXRα, i.e., Nr1h3, an important regulator of cholesterol and FA homeostasis including effects on ABC transporters in macrophages^18^, influencing cholesterol efflux capacity, showed decreased mRNA expression in BMDM of *Apoe*^*−/−*^*Neil3*^*−/−*^ mice (Fig. 5C). In contrast, the BMDM cholesterol loading capacity (Fig. 5D), the expression of the scavenger receptors *Olr1* and *Msr1* (i.e., orthologs of human *LOX-1* and *SR-A1*) were similar, while *Cd36* was decreased in *Apoe*^*−/−*^*Neil3*^*−/−*^ mice (Fig. 5E). In addition to an unfavorable lipid profile, these data suggest that the accelerated atherogenesis in *Apoe*^*−/−*^*Neil3*^*−/−*^ mice could be explained by decreased macrophage cholesterol efflux capacity potentially reflecting alteration in ABC transporters and Nr1h3 expression.

### Serum from *Apoe* ^*−/−*^ *Neil3* ^*−/−*^ mice shows no alteration in cholesterol acceptor capacity

The cholesterol acceptor capacity of serum, a surrogate measure of HDL function was examined in both genotypes by loading mouse macrophages (RAW 264.7 cells) with oxLDL and ^14^C-cholesterol, before incubating these cells with serum from both genotypes. Measurement of the fractional efflux of ^14^C-cholesterol from macrophages revealed similar cholesterol acceptor capacities in the two genotypes (Fig. 5F). Congruent with these results, Lipoprint analysis showed no difference in the lipoprotein subfraction of small HDL particles (Fig. 5G), being the most efficient mediators of cholesterol efflux^19^. Thus, in contrast to the cholesterol efflux capacity in macrophages, the ability of HDL to “accept” cholesterol from macrophages seems not to be impaired in *Apoe*^*−/−*^*Neil3*^*−/−*^ mice.

### No change in genome integrity and accumulation of oxidative DNA damage in Apoe^*−/−*^Neil3^*−/−*^ mice

**Figure 6:**
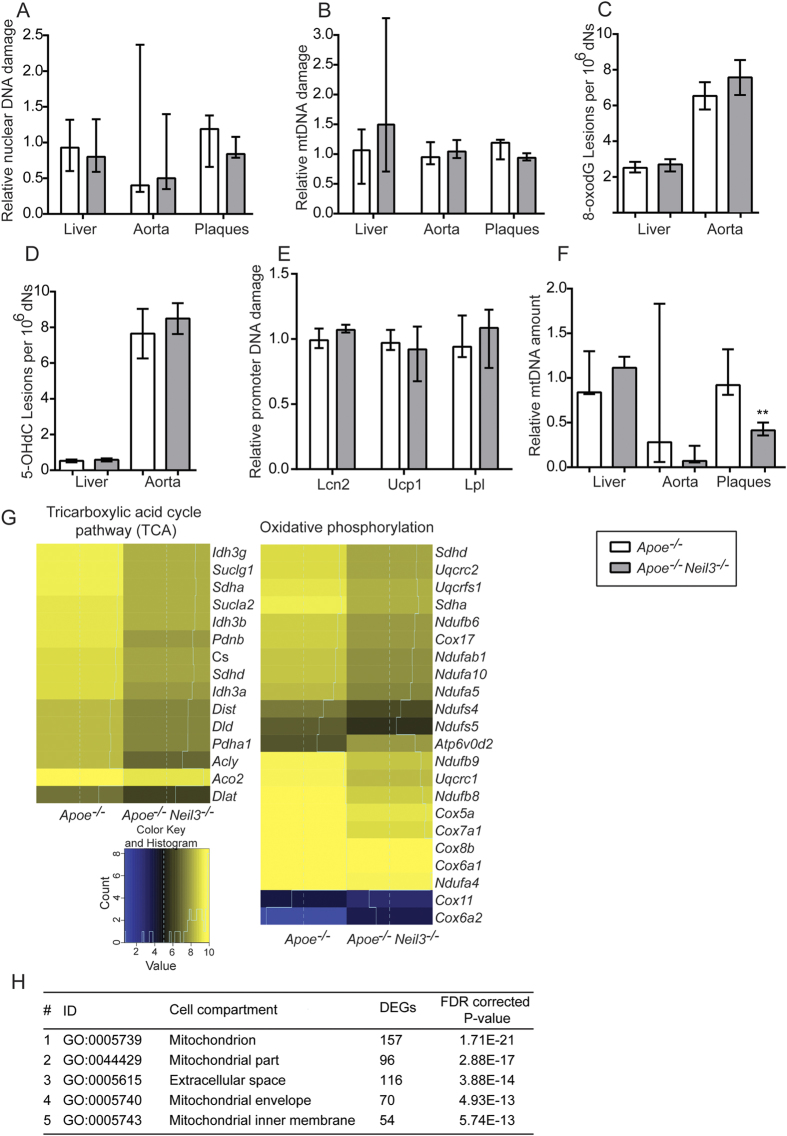
Similar levels of DNA damage but mtDNA copy number analysis and RNA sequencing suggest a reduction in lesional mitochondrial activity in *Apoe*^*−/−*^*Neil3*^*−/−*^ mice. (**A**) Relative nuclear DNA damage and (**B**) relative mitochondrial DNA damage as evaluated by a qPCR-based method in liver (n = 7–9), aorta (n = 5–7) and plaques (n = 6–7). Values are normalized to nuclear and mitochondrial DNA damage in *Apoe*^*−/−*^ mice, respectively. (**C,D**) Accumulation of 8-oxodG and 5-OHdC in liver (n = 6–9) and aortas (n = 3–4), as evaluated by mass spectrometry. (**E**) Relative DNA damage level of promoter regions from *Lcn2*, *Ucp1* and *Lpl* as evaluated by a qPCR-based method in liver (n = 8–9). Values are normalized to promoter DNA damage level in *Apoe*^*−/−*^ mice. (**F**) MtDNA copy number in liver (n = 7–9), aorta (n = 5–7) and plaques (n = 6–7) relative to the mtDNA levels in respective tissues of *Apoe*^*−/−*^ mice. In (**C,D**), aortic data are presented as mean ± SEM and were analyzed using Students’s *t* test. All other data in (**A–F**) are presented as median and interquartile range and were analyzed using Mann-Whitney U test; **p < 0.01. (**G**) Mitochondrial pathways significantly enriched in differentially expressed genes (DEGs) in aorta of *Apoe*^*−/−*^ and *Apoe*^*−/−*^*Neil3*^*−/−*^ mice, as evaluated by RNA sequencing. (**H**) Gene ontology (GO) enrichment analyses of DEGs showing an overrepresentation of genes associated with mitochondrial compartments in *Apoe*^*−/−*^*Neil3*^*−/−*^ as compared to *Apoe*^*−/−*^ mice.

The increased lipid accumulation within the plaques of *Apoe*^*−/−*^*Neil3*^*−/−*^ mice could potentially induce accumulation of DNA damage within the atherosclerotic lesions^20^. Since Neil3 is acknowledged as a DNA repair enzyme that removes oxidative base lesions^21^, we examined if Neil3 deficiency could impair genome integrity or increase bulk accumulation of oxidative DNA base lesions within the plaques of the brachiocephalic artery (BCA) as well as in aorta and liver. A qPCR-based method was used for assessment of nuclear and mitochondrial (mt) DNA integrity, while the bulk level of oxidized DNA base lesions (8-oxoguanine [8-oxodG] and 5-hydroxycytosine [5-OHdC]), including both nuclear and mitochondrial lesions, were measured using mass spectrometry. Surprisingly, none of the examined tissues showed any genotypic differences in nuclear (Fig. 6A) or mitochondrial (Fig. 6B) genome integrity or bulk accumulation of oxidative DNA damage (Fig. 6C,D), of which 5-OHdC is a substrate for Neil3^21^. DNA damage accumulating at gene-regulatory regions (e.g., promoters) can alter gene expression^22^, and the global level of oxidative base lesions may mask accumulation of DNA base modifications at promoter regions in *Apoe*^*−/−*^*Neil3*^*−/−*^ mice. The hepatic genes *Lcn2*, *Ucp1* and *Lpl* that were (i) differentially regulated between the two genotypes and (ii) involved in lipid metabolism (RNA sequencing) were selected for quantitation of promoter DNA damage level using a qPCR-based method. We found no significant differences in the promoter DNA damage levels of *Apoe*^*−/−*^*Neil3*^*−/−*^ relative to *Apoe*^*−/−*^ mice for any of the selected genes (Fig. 6E). However, DNA base modifications important for gene regulation may occur outside of the examined gene-region. In contrast to the lack of changes in oxidative DNA damage in the nucleus, the atherosclerotic plaques of the BCA, but not the non-atherosclerotic thoracic aorta and the liver, showed reduced mitochondrial copy number in *Apoe*^*−/−*^*Neil3*^*−/−*^ mice as compared to *Apoe*^*−/−*^ (Fig. 6F).

### Altered expression of mitochondrial biogenesis genes in *Apoe* ^*−/−*^ *Neil3* ^*−/−*^ mice

To examine differences in the aortic transcriptome between *Apoe*^*−/−*^*Neil3*^*−/−*^ and *Apoe*^*−/−*^ we performed RNA sequencing. This analysis demonstrated that genes involved in the mitochondrial tricarboxylic acid cycle (TCA) pathway and oxidative phosphorylation clearly was downregulated in Neil3 deficient mice (Fig. 6G). Also, gene ontology enrichment analyses of aortic DEGs revealed a significant overrepresentation of genes associated with the mitochondrial compartments in *Apoe*^*−/−*^*Neil3*^*−/−*^ mice (Fig. 6H). Thus, the accelerated atherogenesis in the *Apoe*^*−/−*^*Neil3*^*−/−*^ mice appeared not to be associated with impaired plaque DNA stability, but with alterations in mitochondrial functions in the macroscopically non-atherosclerotic thoracic aorta and reduced mitochondrial copy numbers in the atherosclerotic BCA.

### Neil3 deficiency alone shows no effects on lipid parameters in serum

Finally, we examined if Apoe deficiency is required for the involvement of Neil3 in lipid metabolism by analyzing fasting serum lipid profile in wild type (Wt) and *Neil3*^*−/−*^ mice fed a high-fat diet for 14 weeks. As shown in Supplemental Fig. S6, serum TG and total cholesterol showed no differences between Wt and Neil3-deficient mice, and in general, mice on an *Apoe*^*−/−*^ background had higher levels of these lipid parameters and in particular *Apoe*^*−/−*^*Neil3*^*−/−*^ mice. Thus, it seems that the effect of Neil3 deficiency, at least for the serum lipid profile, is dependent on an environment characterized by high lipid levels and metabolic stress as in an Apoe-deficient phenotype.

## Discussion

**Figure 7:**
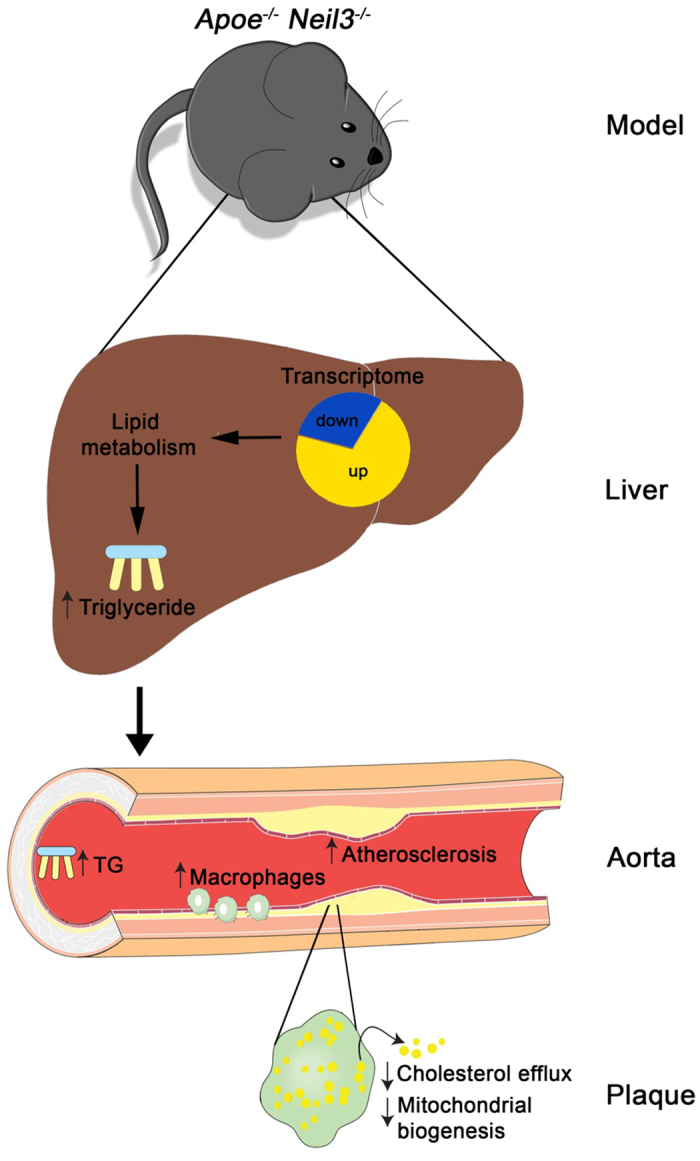
Neil3 deficiency promotes atherosclerosis in *Apoe*^*−/−*^ mice on a high-fat diet through effects on lipid metabolism and macrophages, potentially involving non-canonical effects. *Neil3*^*−/−*^*Apoe*^*−/−*^ mice show enhanced liver steatosis with increased TG levels, potentially caused by increased substrate availability (MUFA). Transcriptome analysis revealed marked alterations in hepatic pathways involved in lipid metabolism. The hepatic accumulation of lipids in *Apoe*^*−/−*^*Neil3*^*−/−*^ mice results in a hyperlipidemic plasma profile with subsequent enhanced atherosclerosis. Within the atherosclerotic lesion there is an accumulation of macrophages indicating increased lesional proliferation. The macrophages in *Neil3*^*−/−*^*Apoe*^*−/−*^mice show attenuated cholesterol efflux capacity, further enhancing the metabolic stress within the plaque, contributing to altered mitochondrial biogenesis. The authors wish to acknowledge SERVIER Medical Art (www.servier.fr) for use of their medical art kits when making the illustration.

Based on biochemical characterization, NEIL3/Neil3 is acknowledged as a DNA repair enzyme initiating repair of oxidized DNA base lesions^21^. Recent reports have also indicated a role of NEIL3/Neil3 in maintenance of cerebral stem and progenitor cells by counteracting oxidative stress-induced DNA damage^7^,^9^. The biological function(s) of NEIL3/Neil3 are, however, still elusive. Here, we examined the role of murine Neil3 in mice prone to develop atherosclerosis. When challenged with a high-fat diet, male *Apoe*^*−/−*^*Neil3*^*−/−*^ mice displayed increased atherosclerosis as compared to *Apoe*^*−/−*^ controls, resulting from an unfavorable lipid profile including increased hepatic TG levels and reduced macrophage cholesterol efflux capacity (Fig. 7). The different phenotypes were not related to altered genomic stability or global accumulation of oxidized DNA damage, and although we have no firm evidence, it is tempting to hypothesize a role for Neil3 beyond genome-wide canonical repair.

Increasing evidence supports that DNA glycosylases are involved in regulating energy metabolism. Recently, Ogg1-deficient mice fed high-fat diet displayed increased adiposity and hepatic steatosis^23^. Also, Neil1-deficient mice developed severe obesity, fatty liver disease and dyslipidemia^24^. Here, we show that Neil3 influences lipid metabolism on an Apoe-deficient, but not on a Wt background. The *Apoe*^*−/−*^*Neil3*^*−/−*^ mice showed increased plasma levels of TG, LDL-cholesterol and oxLDL and accelerated hepatic steatosis with increased TG accumulation and altered hepatic FA distribution. Also, mRNA sequencing showed marked alterations in livers from *Apoe*^*−/−*^*Neil3*^*−/−*^ mice, affecting several pathways involved in lipid metabolism, with increased FA/MUFA-driven TG synthesis as a potential mechanism for the accumulation of TG in liver and plasma. Indeed, endogenously synthesized MUFA are suggested to serve as main substrates for the synthesis of TG and cholesterol esters^25^. Also, C18:1n-9 level, which constitutes one of the preferred substrates for enzymes responsible for TG and cholesteryl ester synthesis^26^, was increased in the liver from the *Apoe*^*−/−*^*Neil3*^*−/−*^ mice. The hepatic lipid accumulation in *Apoe*^*−/−*^*Neil3*^*−/−*^ mice, however, could also involve other mechanisms such as altered uptake and secretion of FA^27^. Also, whether the hepatic changes are directly related to Neil3 deficiency with effects at specific gene regulatory regions, or if they rather involve indirect effects via e.g. FA-dependent regulation of hepatic transcription factors^28^, is presently not known.

Our finding of accelerated atherogenesis in *Apoe*^*−/−*^*Neil3*^*−/−*^ mice was accompanied by enhanced aortic *Cd68* expression and increased absolute number of plaque macrophages. RNA sequencing of thoracic aorta further suggested a role for macrophage/lipid interaction in the accelerated atherosclerosis in the *Apoe*^*−/−*^*Neil3*^*−/−*^ mice. Indeed, macrophages from *Apoe*^*−/−*^*Neil3*^*−/−*^ mice displayed an increased proliferative response upon stimulation with modified lipids, potentially contributing to increased plaque accumulation of macrophages. We have previously reported that Neil3-deficient mice show reduced proliferation of neural stem progenitor cells after cerebral hypoxic ischemia^9^. Our findings herein could suggest tissue- and stressor-specific influences of the interaction between Neil3 and cell proliferation. Moreover, Neil3 deficiency reduced the cholesterol efflux capacity of macrophages by downregulating ABC transporters. Thus, in addition to an unfavorable lipid profile and an increased lipid-induced macrophage proliferation, an attenuated cholesterol efflux in plaque macrophages may have contributed to accelerated atherogenesis in *Apoe*^*−/−*^*Neil3*^*−/−*^ mice.

Neil3 shows overlapping substrate specificity with the DNA glycosylases Neil1, Neil2 and Nth1. Despite these backup activities, our results indicate that Neil3 is indispensable to avoid increased atherosclerosis in mice on an *Apoe*^*−/−*^ background. Several studies have showed that the substrate specificity of DNA glycosylases is not only dependent on the nucleobase sequence, but also on DNA structure. Wallace and coworkers demonstrated that NEIL3, but not NEIL1, NEIL2, NTH1 or OGG1 can remove thymine glycol in quadruplex DNA *in vitro*^29^. NEIL1, NEIL2 and NEIL3 are also shown to remove oxidized guanine lesions from quadruplex DNA structures formed by promoter and telomere sequences^30^,^31^. Altogether, growing evidence suggests that NEIL glycosylases may have gene regulatory functions. Thus, it is plausible that the atherogenic lipid profile observed in the *Apoe*^*−/−*^*Neil3*^*−/−*^ mice could be caused by deficient Neil3 activity at promoters regulating TG and cholesterol metabolism. Assessment of genome integrity and total genome-wide oxidative DNA base damage by mass spectrometry showed no genotypic differences in the examined tissues relevant for lipid metabolism and atherogenesis. However, a Neil3-dependent accumulation of oxidative base lesions in *Apoe*^*−/−*^*Neil3*^*−/−*^ mice at certain promoter regions, beyond those that were examined in the present study, will most likely be masked by the bulk levels of oxidative base lesions measured by mass spectrometry. Thus, the effects of Neil3 deficiency in our Apoe-deficient model on high-fat diet, characterized by lipid and metabolic stress, could still involve induction of oxidative base lesions. In order to further address these issues, we need methods detecting oxidative lesions at single base resolution.

Epigenetic DNA methylation represents a major mechanism to regulate gene expression, in which DNA methyltransferases methylate cytosine at the 5-carbon position to generate 5-methyl cytosine (5-mC). Active demethylation of 5-mC involves numerous enzymes, including ten-eleven translocation enzymes and the thymine DNA glycosylase^32^. Several reports show that epigenetic DNA methylation is associated with plasma lipid concentrations and increased cardiovascular risk^33^,^34^,^35^. Recently, Vermeulen and coworkers showed that several DNA glycosylases, including Neil3, are epigenetic readers of oxidized derivatives of 5-mC^36^. Based on the findings in the present study, a possible role of Neil3 in active demethylation of 5-mC in promoters regulating lipid metabolism, should be explored in future experiments.

Genes involved in the mitochondrial TCA cycle pathway and oxidative phosphorylation were downregulated in non-atherosclerotic aortic tissue of *Apoe*^*−/−*^*Neil3*^*−/−*^ mice, suggesting that decreased mitochondrial function could be an early feature of atherogenesis in this genotype. This feature was further supported by the reduced plaque mtDNA copy number. Recently, another study has suggested a crucial role of damaged mitochondria in atherogenesis, linking impaired mitochondrial function and reduced oxidative phosphorylation to increased atherogenesis in *Polg*^*−/−*^*Apoe*^*−/−*^ mice^37^. In support of the nuclear location of Neil3^38^, the mitochondria-related genes investigated in our study as well as known regulators of mtDNA copy number, are all encoded by nuclear DNA^39^, suggesting that our observed alteration in plaque mtDNA copy number most probably reflects indirect effects of Neil3 deficiency, such as metabolic or oxidative stress within the plaque (Fig. 7).

We have recently shown that a certain genetic variant in *NEIL3* (i.e., *NEIL3* rs12645561; TT genotype) was associated with increased risk of MI^40^. Herein, we report marked upregulation of *NEIL3* expression in carotid atherosclerosis as compared to non-atherosclerotic vessels in two separate cohorts. In the Oslo cohort, we found a significant positive correlation between plaque expression of *NEIL3* and the macrophage marker *CD68*, and modified lipids enhanced the *NEIL3* expression of in macrophages. Finally, PBMC from patients with carotid atherosclerosis did not show increased *NEIL3* expression, suggesting that *NEIL3* is upregulated in macrophages within the atherosclerotic lesion as a response to lipid exposure. Taken into account our findings in the experimental models, we hypothesize that lipid-induced upregulation of *NEIL3* within plaques could represent a counteracting response to unfavorable lipid profiles and metabolic stress in patients with atherosclerotic disorders. Hence, studies in a mouse model overexpressing Neil3 may provide valuable insight into the role of Neil3 in human atherosclerosis.

In summary, our results reveal a novel role for murine Neil3 in balancing lipid metabolism that involves effects on hepatic lipid metabolism as well as cholesterol efflux capacity in macrophages, resulting in accelerated atherosclerosis in *Neil3* deficient mice on an *Apoe*^*−/−*^ background (Fig. 7). We propose that Neil3/NEIL3 could be involved in regulation of transcriptional network(s) responding to lipid stress via removal of oxidative lesions at gene-specific sequences of importance for promoter activity and that this also could involve activation of non-canonical pathways.

## Experimental Procedure

An expanded version of the Experimental Procedure section is available in the Online Data Supplementary section.

### Ethics

The BiKE study was performed after approval by the Ethical Committee of Northern Stockholm (file numbers 02/147 and 2009/295-31/2) and was in compliance with institutional guidelines. Establishment and handling of the Oslo Biobank were approved by the Regional Committee for Medical and Health Research Ethics for South-East Norway (project number 6.2009.549, ref.no 2009/5237, 2009/613, 2014/2078, and ref.no S-06172). Both the BiKE study and the Oslo Biobank protocols were in agreement with the principles of the Declaration of Helsinki and signed informed consent was obtained from all individuals. The murine studies were according to Norwegian law regulating animal experiments and international treaties ratified by Norway. All experiments were approved by the Norwegian Animal Research Committee.

### Human subjects and tissue sampling from human subjects

BiKE *(Biobank of Karolinska Endarterectomies)* provided human samples for microarray experiments. Briefly, endarterectomy specimens from the carotid artery (n = 106) were collected from patients diagnosed with >70% carotid artery stenosis at Department of Vascular Surgery of the Karolinska Hospital from 2001 to 2008 (Supplementary Table S1). Aortic and iliac non-atherosclerotic arteries obtained from organ donors (n = 10) were used as negative controls. The samples from the *Biobank of Oslo* consisted of carotid plaques obtained from patients with internal carotid artery stenosis (≥70%) undergoing carotid endarterectomy (n = 68) at Oslo University Hospital Rikshospitalet (Supplementary Table S2). Iliac non-atherosclerotic arteries obtained from organ donors (n = 9) were used as negative controls.

### Animals

Neil3-deficient mice were generated by germline deletion of exons 3–5 and backcrossed into C57BL/6 mice for 10 generations. *Apoe*^*−/−*^*Neil3*^*−/−*^ mice were generated by crossing *Neil3*^*−/−*^ mice with *Apoe*^*−/−*^ (C57BL/6 background) mice, obtained from Taconic. The mice were fed high-fat diet (R638, Lantmännen, Sweden; 21% fat by weight [62.9% saturated, 33.9% unsaturated and 3.4% polyunsaturated], 17.2% protein, 43% carbohydrates and 0.15% cholesterol) *ad libitum* from 8 weeks of age until harvesting at 26 weeks of age.

### Statistical analysis

Except for the RNA sequencing analysis and experiments with a total sample size of n ≤7, all data were analyzed using non-parametric tests, i.e., the Mann–Whitney U test, Spearman’s rank correlation or Kruskal-Wallis test with Dunn’s post test for multiple comparisons. Experiments with a total sample size of n ≤7 were analyzed using two-tailed unpaired Student’s *t* test and categorical data were analyzed using the chi-square test. Data are presented as median and interquartile range unless otherwise stated. P values <0.05 were considered statistically significant.

## Additional Information

**How to cite this article**: Skarpengland, T. *et al*. Neil3-dependent base excision repair regulates lipid metabolism and prevents atherosclerosis in Apoe-deficient mice. *Sci. Rep.* **6**, 28337; doi: 10.1038/srep28337 (2016).

## Supplementary Material

Supplementary Information
