# Supplementary material for: Neil3-dependent base excision repair regulates lipid metabolism and prevents atherosclerosis in Apoe-deficient mice
Source: Sci Rep. 2016 Jun 22;6:28337. doi: 10.1038/srep28337 (PMC4916448; doi:10.1038/srep28337)
Supplement: Supplementary Information [file srep28337-s1.pdf]

Supplemental Data

Neil3-dependent base excision repair regulates lipid metabolism and prevents atherosclerosis in Apoe-deficient mice

Tonje Skarpengland, MD,PhD[1,11]; Sverre Holm, MSc,PhD[1]; Katja Scheffler, MSc,PhD[6,12,14]; Ida Gregersen, MSc,PhD[1,11]; Tuva B. Dahl, MSc,PhD[1,11,13]; Rajikala Suganthan, MSc[7]; Filip M. Segers, MSc,PhD[1]; Ingunn Østlie, MSc[8]; Jeroen J.T. Otten, MSc,PhD[20]; Luisa Luna, MSc,PhD[7]; Daniel F.J. Ketelhuth, PhD[19]; Anna M. Lundberg, PhD[19]; Christine G. Neurauter, MSc[7]; Gunn Hildrestrand, MSc,PhD[7]; Mona Skjelland, MD,PhD[2]; Bodil Bjørndal, MSc,PhD[17]; Asbjørn M. Svardal, MSc,PhD[17]; Per O. Iversen, MD,PhD[5,10,12]; Ulf Hedin, MD,PhD[20]; Ståle Nygård, PhD[14]; Ole K. Olstad, PhD[9]; Kirsten Krohg-Sørensen, MD,PhD[4,11]; Geir Slupphaug, MSc,PhD[15,16]; Lars Eide, PhD[6,11]; Anna Kuśnierczyk, MSc,PhD[15,16]; Lasse Folkersen, MSc, PhD[22]; Thor Ueland, PhD[1,11,13]; Rolf K. Berge, MSc,PhD[17,18]; Göran K. Hansson, MD,PhD[19]; Erik A.L. Biessen, PhD[21]; Bente Halvorsen, MSc,PhD,[1,11,13,#†]; Magnar Bjørås, MSc[7,11,16,#]; Pål Aukrust, MD,PhD[1,3,11,13,#]

[1]Research Institute of Internal Medicine, [2]Department of Neurology, [3]Section of Clinical Immunology and Infectious Diseases, [4]Department of Thoracic and Cardiovascular Surgery, [5]Department of Hematology, [6]Department of Medical Biochemistry, [7]Department of Microbiology, Oslo University Hospital Rikshospitalet, [8]Department of Pathology, Oslo University Hospital Radiumhospitalet, [9]Department of Medical Biochemistry, Oslo University Hospital Ullevål, [10]Department of Nutrition, [11]Institute of Clinical Medicine, [12]Institute of Basic Medical Research, [13]K.G. Jebsen Inflammatory Research Center, [14]Department of Informatics, University of Oslo, Oslo, [15]Department of Cancer Research and Molecular Medicine, [16]PROMEC

Core Facility for Proteomics and Metabolomics, Norwegian University of Science and Technology, Trondheim, [17]Department of Clinical Science, University of Bergen, [18]Department of Heart Disease, Haukeland University Hospital, Bergen, Norway, [19]Center for Molecular Medicine, [20]Department of Surgery, Karolinska University Hospital, Stockholm, Sweden, [21]Department of Experimental Vascular Pathology, University of Maastricht, Maastricht, The Netherlands, [22]Center for Biological Sequence Analysis, Technical University of Denmark, Copenhagen, Denmark.

[#]Contributed equally.

Corresponding author: Bente Halvorsen, Research Institute of Internal Medicine, Oslo University Hospital, 0424 Oslo, Norway; Phone: +4723070000; Fax: +4723073630; Email: Bente.Halvorsen@rr-research.no

Supplemental Material and Methods

Human subjects and tissue sampling from human subjects. BiKE *(Biobank of Karolinska Endarterectomies)* provided human samples for microarray experiments. Details of the BiKE study has been described previously[1,2]. Briefly, endarterectomy specimens from the carotid artery (n=106) were collected from patients diagnosed with >70% carotid artery stenosis at Department of Vascular Surgery of the Karolinska Hospital from 2001 to 2008 (Supplemental Table 1). Endarterectomy specimens were snap frozen and RNA was extracted as previously described[3]. Aortic and iliac arteries obtained from organ donors (n=10) were used as negative controls. The samples from the *Biobank of Oslo* consisted of carotid plaques obtained from patients with internal carotid artery stenosis (≥70%) undergoing carotid endarterectomy (n=68) at Oslo University Hospital Rikshospitalet. Details regarding patient exclusion and inclusion criteria, as well as patient examination, diagnosis and follow-up are described elsewhere[4,5]. Briefly, patients were classified into symptomatic (n=54) and asymptomatic (n=14) categories according to presence or absence of relevant clinical symptoms (i.e., transient ischemic attack, amaurosis fugax or stroke ipsilateral to the stenotic internal carotid artery) within the last 6 months before surgery (Supplemental Table 2). Common iliac arteries collected during organ transplantation from 9 donors without cardiovascular disease were used as negative controls. Atherosclerotic carotid plaques and control samples were snap frozen and stored at -80 °C until use. PBMC obtained from 15 patients (7 females and 8 males, median age 61.5 years, IQR 57-79 years) undergoing carotid endarterectomy were collected within 2 days before surgery, as previously described[6]. For comparison, PBMC were also collected from 16 apparently healthy (based on disease history, clinical examination and normal levels of CRP) blood donors (3 females and 13 males, median age 69 years, IQR 66-70 years) recruited from the same region in Norway.

BiKE gene expression microarray. RNA was hybridized to Affymetrix HG-U133 plus 2.0 A Genechip® arrays according to standard protocol (http://www.affymetrix.com) and mRNA levels were measured and analyzed at the Karolinska Institute Affymetrix microarray core facility as previously described[2,7]. Microarray data were pre-processed, normalized and log2-transformed using the RMA algorithm[8]. The data set is available from Gene Expression Omnibus, accession number GSE21545.

THP-1 cells. The human monocytic cell line THP-1 (American Type Culture Collection, Rockville, MD) was grown in RPMI 1640 (PAA Laboratories, Pasching, Austria), containing 1% penicillin, 10% FCS, 0.1% gentamycin (Sigma-Aldrich, St Louis, MO) at 37 °C with 5 % CO_2. The cells were differentiated into macrophages by incubation with phorbol myristate acetate (PMA, 100 nM, Sigma Aldrich) for 24 hours. Thereafter, the cells were stimulated with endotoxin-free VLDL (25 μg/ml; Calbiochem, Darmstadt, Germany) and cholesterol crystals (100 μg/ml), prepared as previously described[9], for 6 hours before harvesting of cell pellets.

Animals. Neil3-deficient mice were generated by germline deletion of exons 3–5 as previously described[10] and backcrossed into C57BL/6 mice for 10 generations. *Apoe*$^{-/-}$*Neil3*$^{-/-}$ mice were generated by crossing *Neil3*$^{-/-}$ mice with *Apoe*$^{-/-}$ (C57BL/6 background) mice, obtained from Taconic. All mice included in the study were born at Centre for Comparative Medicine, Oslo University Hospital Rikshospitalet. Only male mice were included in the study. The mice were fed high-fat diet (R638, Lantmännen, Sweden; 21% fat by weight [62.9% saturated, 33.9% unsaturated and 3.4% polyunsaturated], 17.2% protein, 43% carbohydrates and 0.15% cholesterol) *ad libitum* from 8 weeks of age until harvesting at 26 weeks of age.

Murine tissue collection and blood sampling. Mice were anaesthetized under non-fasting conditions with intraperitoneal injection of 0.1 ml per 10 g body weight Hypnorm/Dormicum (1.25 mg/ml Dormicum, 2.5 mg/ml Fluanisone, 0.079 mg/ml Fentanyl citrate) and killed by

exsanguination. Blood was collected at sacrifice by trans-thoracic cardioscentesis using a 1ml syringe without (serum) or with (plasma) coating of 0.5 M EDTA (Fluka, Sigma-Aldrich). Serum was obtained after coagulation at room temperature for 30 minutes before centrifugation at 1000*g* for 15 minutes. EDTA blood was immediately placed on ice and centrifuged within 30 minutes at 2000*g* (4 °C) for 20 minutes to obtain platelet-poor plasma. Serum and plasma were aliquoted and kept at -80°C until use. For collection of tissue specimens, the vasculature was perfused through the left ventricle with 2 ml of sterile PBS at a rate of approximately 1 ml/minute. The cranial half of the heart was placed in OCT compound (Tissue-Tek, Sakura Finetek, Torrance, CA) and frozen for subsequent cryo-sectioning. Thoracic aortas were either placed on 4% paraformaldehyde (PFA) in PBS for *en face* quantification of atherosclerosis, or snap frozen together with the other organs for subsequent analysis. For plaque-analyses, the proximal brachiocephalic artery, containing large occluding plaques, was excised and snap frozen.

Histological and morphometric analyses of the aorta and aortic root. The thoracic aorta was fixed in 4% PFA in PBS and dissected under an Olympus SZX12 microscope. All adventitial fat tissue was carefully resected. The aortic arch was longitudinally cut open, pinned on a black math and stained with Sudan IV (Sigma-Aldrich). *En face* plaque area was quantified (plaque surface area/total surface area of aortic arch × 100) by using identical denominator (proximal 16.5 mm^2 area of the aortic arch) in all mice (excluding the branching vessels). Frozen hearts in OCT compound (Tissue-Tek, Sakura Finetek, Torrance, CA) were serially sectioned towards the base of the heart on a cryostat. 10 μm frozen sections were collected in cranial direction, starting at 100 μm distance after appearance of the aortic cusps. Sections were air-dried and fixated with either 4% PFA or cold acetone. PFA-fixed sections were stained with Oil Red O (Sigma-Aldrich) and hematoxylin (Vector Laboratories Inc., Burlingame, CA) and relative lesion areas (plaque

area/vessel area inside external elastic lamina × 100) were calculated in 8 consecutive sections at 100 μm intervals. The mean relative lesion area was calculated from 8 sections from each mouse. This method is reported to reduce the chance of miscalculation of lesion areas due to oblique sections[11]. Necrotic core was assessed in sections from the aortic root stained with hematoxylin (Vector Laboratories Inc.) and eosin (Histolab, Gothenburg, Sweden) and defined as acellular areas with or without cholesterol crystals. Necrotic core was quantified twice and mean values were used for analyses. Total collagen content, thin vulnerable fibers (green), and mature collagen (red), as assessed by the fraction: "collagen area/plaque area within internal elastic lamina × 100", was stained with Picrosirius Red (Histolab). Images were captured using both bright field and polarized microscopy. Pixels of a specific red hue in bright field images were interpreted as collagen and counted with Fiji, an ImageJ-derived imaging software. Green and red fibers were isolated by using their colorimetric footprint in the polarized light microscopy images and analyzed using the mentioned software. Plaque cell content was assessed by application of primary rat anti-mouse antibodies for Vcam1 (Pharmingen, San Diego, CA), Cd68 (AbD Serotec, Kildington, UK), Cd4 (Pharmingen), Cd8 (Pharmingen), and rabbit anti-mouse antibody SMC α-actin (Abcam, Cambridge, UK) to acetone-fixed sections. These quantifications are shown as both % positive staining of total lesion area (positive staining area/area inside lamina elastic interna × 100) and absolute positive stained area as mm^2 for Cd68, Vcam1 and SMC α-actin, whereas Cd4 and Cd8 are shown as both number of positive cells per lesion (number of positive cells/area inside lamina elastic interna) and as number of cells related to area (positive cells/mm^2). Images were captured by use of a Nikon DS Fi1 camera on a Nikon SMZ1500 microscope and a Nikon Eclipse E400 microscope. All analyses were performed blindly by one person using NIS Elements BR3.1 software and Fiji software.

Liver histology. To examine liver histology, livers were fixed in formalin and embedded in paraffin and then cut into 5 µm sections. Sections were deparaffinized and stained with hematoxylin (Vector Laboratories Inc.) and eosin (Histolab). Images were captured by use of a Nikon DS Fi1 camera and a Nikon Eclipse E400 microscope.

Murine plasma glucose and plasma and liver lipids. Liver and plasma lipids were extracted in conformity with Bligh and Dyer[12], evaporated under nitrogen and redissolved in isopropanol. Subsequent analyses of liver and plasma lipids were measured enzymatically by use of the Hitachi 917 system (Roche Diagnostics GmbH, Mannheim, Germany), using commercial kits[13]. In the fasting condition, serum cholesterol and TG levels were quantified by use of enzymatic colorimetric kits (Wako Chemical GmbH, Neuss, Germany). Plasma levels of non-esterified FA were measured on the Hitachi 917 system using a commercially available FFA kit (NEFA C, Wako Chemicals GmbH). Plasma oxLDL was measured by plotting relative light units from a luminometer against a standard curve with known concentrations according to manufacturer's protocol (MyBiosource, San Diego, CA). Fasting glucose levels were measured with a glucometer (Accu-Chek Aviva, Roche, Basel, Switzerland).

Murine plasma and liver FA composition. Lipids were extracted using a mixture of chloroform and methanol. The extracts were trans-esterified using BF_3 (boron trifluride)-methanol. To remove neutral sterols and non-saponifiable material, extracts of fatty acyl methyl esters were heated in 0.5 M KOH in an ethanol-water solution (9:1). Recovered FAs were re-esterified using BF_3-methanol. The methyl esters were quantified by gas chromatography as previously described[14].

Murine lipoprotein subfractions. Lipoprotein subfractions in plasma were measured by a linear polyacrylamide gel electrophoresis system. Lipoprint HDL kit and Lipoprint LDL kit

(Quantimetrix Corporation, Redondo Beach, CA) were prepared according to manufacturer's protocol. The gels were scanned and analyzed using Lipoware software (Quantimetrix).

Murine plasma carnitine derivatives. Carnitine derivatives were measured in plasma using LC-MS/MS as previously described[15], with some modifications of the HPLC conditions[16,17].

Cytokine measurements. Murine serum cytokines were quantified by use of Bio-Plex Pro Mouse Cytokine 23-plex (Bio-Rad Laboratories, Hercules, CA) and analyzed on a Multiplex analyzer (Bio-Plex 100, Bio-Rad Laboratories).

Activities of murine liver enzymes involved in FA metabolism. Details are described elsewhere[13]. Briefly, the liver samples were homogenized and nuclei in cell debris were removed by centrifugation at 750*g* for 15 minutes at 4 °C. The post-nuclear fraction was removed and used for further analysis. The assays for Cpt-1 (in the presence and absence of 15 μM malonyl-CoA) and Cpt-2 were performed according to Bremer et al.[18], with some modifications[19]. The activities of Fas and Gpat were measured in the post-nuclear fraction, as described previously[20], with some modifications[19]. Acc was measured in the post-nuclear fraction as described by Skorve et al.[20].

Isolation of murine BMDM. Bone marrow cells were isolated by flushing femurs and tibias with sterile ice-cold PBS. Cells from each mice were kept separately and kept in RPMI 1640 (PAA Laboratories, Cölbe, Germany) containing 10% heat–inactivated FCS, 100 U/ml penicillin, 100 μg/ml streptomycin, 2 mM L-glutamine, 10 mM HEPES (all from Sigma Aldrich), and supplemented with 15% L929–conditioned medium to generate BMDM[21]. Medium was replaced every three days. After 7-8 days, differentiated BMDMs were either used for *in vitro* assay (see below) or frozen in liquid nitrogen.

Murine BMDM cholesterol efflux and loading capacity. After 7 days of differentiation, BMDM were split and plated into 24-wells dishes and lipid loaded by incubation with oxLDL (20 μg/ml) in regular growth medium with 15% L-929 medium plus 0.5 μCi/ml ^{14}C-cholesterol

(American Radiolabel Chemicals, Saint Louis, MO), dissolved in ethanol. After 48 hours, the radiolabeled media was removed, and the foam cells were washed twice with 0.2% BSA in RPMI 1640 (wt:v). Thereafter, the cells were incubated in RPMI 1640 with 2.5% heat-inactivated mouse serum (pooled serum from 4 C57BL/6 wild type mice) as cholesterol acceptor. After 4 hours, the cell medium was collected and the cells were harvested in 0.2 mol/l NaOH. The radioactivity was measured by liquid scintillation counting using TRI-CARB 2300 TR Scintillation Counter (Packard, Waltham, MA). The macrophage loading capacity was calculated as disintegrations per minute (dpm) per total protein as measured using BCA protein detection assay (Pierce, Rockford, IL). Data are presented as fractional (%) cholesterol efflux calculated as dpm (medium)/dpm (medium + cell-associated)] × 100.

Serum cholesterol acceptor function. To assess the cholesterol acceptor capacity of the sera obtained from *Apoe*$^{-/-}$*Neil3*$^{-/-}$ and *Apoe*$^{-/-}$ mice, RAW 264.7 cells were loaded with oxLDL (20 μg/ml) in regular DMEM with 10% FCS plus 0.5 μCi/ml ^{14}C-cholesterol for 48 hours. After 24 hours, 0.3 mM 8-Br-cAMP (Sigma Aldrich) was added and the cells incubated for another 20 hours to boost the *Abca1* levels. The cells were washed, and 2.5% heat-inactivated serum from *Apoe*$^{-/-}$ and *Apoe*$^{-/-}$*Neil3*$^{-/-}$ mice in DMEM was used as ^{14}C-cholesterol acceptor and the fractional efflux was measured as detailed above.

Immunophenotyping of bone marrow aspirates. We enumerated long-term hematopoietic stem cells (LT-HSC) and multipotent progenitor cells (MPPs)[22] by flow cytometric analyses of fresh bone marrow aspirates from *Apoe*$^{-/-}$ (n=10) and *Apoe*$^{-/-}$*Neil3*$^{-/-}$ (n=9) mice after surface labeling with anti-mouse-antibodies against the following surface antigens: Sca1 (clone E13-161.7, Becton Dickinson [BD], San Jose, CA), c-kit (clone 2B8, eBioscience, San Diego, CA), Flt3 (clone A2F10, BioLegend, San Diego, CA), Cd150 (clone TC15-12F12.2, BioLegend), Cd48 (clone HM48-1, BioLegend), as well as Lineage-mix containing: Cd4 (clone RM4-5, BD),

Cd8a (clone 53-6.7, BD), B220 (clone RA3-6B2, BD), Cd5 (clone 53-7.3, BioLegend) Gr-1 (clone RB6-8C5, BioLegend), Cd11b (clone M1/70, BioLegend) and Ter119 (clone TER-119, BioLegend). Viability was assessed by use of DAPI staining (Invitrogen).

Macrophage chemotaxis. Peritoneal macrophages were isolated three days after peritoneal injection of 1ml 3% Brewer's thioglycollate (BD, Franklin Lakes, NJ). The cells were counted using Invitrogen Countess automated cell counter (Invitrogen) as a measure for macrophage migration capacity.

DNA damage and copy number analysis by qPCR. A qPCR-based analysis built on the ability of DNA damage to inhibit restriction enzyme cleavage was performed as previously described[23]. Briefly, tissues were homogenized by using a FastPrep®-24 (MP Biomedical, Santa Ana, CA) and genomic DNA was isolated by using the AllPrep DNA/RNA/Protein Mini Kit (Qiagen, Hilden, Germany), according to manufacturer's protocol. *Taq*$^{\alpha}$I restriction enzyme digestion and subsequent real-time PCR was carried out with 30 ng and 6 ng of total DNA for nuclear and mitochondrial DNA damage detection, respectively. Relative amounts of PCR products were calculated by the comparative Ct method; 2exp-(CtTaqI - Ctnt), where Ct^{TaqI} and Ct^{nt} represent Ct values of TaqI-treated and non-treated genomic DNA, respectively. The following primers were used: *Ndufa9* promoter region forward, *5'* tggtgactcctacctgaagc and reverse, 5' ttcggtcgtgaattttgttt for nDNA and 12S ribosomal RNA gene (*mt-Rnr1*) forward, 5' actcaaaggacttggcggta and reverse, 5' agcccatttcttcccatttc for mtDNA. For detection of promoter DNA damage levels the following primers were used: *Lcn2* forward, 5'acctttaatcccagcactcg and reverse, 5' gtatagccctggctgtcctg, *Lpl* forward, 5' ccatgactttctgcctctga and reverse, 5' tggcaatatgggtctccttc and *Ucp1* forward, 5' tcgcccaactaaaagtgacc and reverse, 5' gagggaatccatgcaaaaac. The relative

mtDNA copy number was calculated by the comparative ΔCt method using the Ct values from non-treated samples of mtDNA and nuclear DNA.

LC-MS/MS analysis of 8-oxoguanine and 5-hydroxycytosine in genomic DNA. Genomic DNA was isolated from human tissue using the AllPrep DNA/RNA/Protein Mini Kit (Qiagen), according to manufacturer's protocol. 2 µg of genomic DNA were enzymatically hydrolyzed to deoxyribonucleosides by incubation in a mixture of DNAse I (Roche, 04716728001) nuclease P1 from *Penicillium citrinum* (Sigma, N8630), and alkaline phosphatase from *E. coli* (Sigma Aldrich, P5931) in 10 mM ammonium acetate buffer, pH 5.3 at 40°C for 30 minutes. Three volume equivalents of ice-cold methanol were added to the reactions after digestion was completed to precipitate proteineous contaminants. Following centrifugation at 16000*g* for 30 minutes the supernatants were collected in new tubes and dried under vacuum at room temperature. The resulting residues were dissolved in 50 µl of water for LC-MS/MS quantification of 8-oxoguanine (8-oxodG) and 5-hydroxydC (5-OHdC). For quantification of unmodified nucleosides (dA, dC, dG, dT) samples were further diluted in water 1:500. Chromatographic separation of nucleosides was performed using a Shimadzu Prominence LC-20AD HPLC system with an Ascentis Express C18 2.7 µm 150 x 2.1 mm i.d. column equipped with an Ascentis Express Cartridge Guard Column (Supelco Analytical, Bellefonte, PA, USA) with EXP Titanium Hybrid Ferrule (Optimize Technologies Inc.) at a flow rate of 0.14 ml/min at ambient temperature. The mobile phase consisted of A (0.1% formic acid in water) and B (0.1% formic acid in methanol). The following conditions were employed during chromatographic separation: unmodified nucleosides – starting with 90% A and 10% B for 0.1 minute, followed by a 2.4 minutes linear gradient of 10-60% B, and 4.5 minutes re-equilibration with the initial mobile phase conditions; 8-oxodG and 5-OHdC - starting with 95% A and 5% B for 0.5 minutes,

followed by a 7.5 minutes linear gradient of 5 - 45% B, and 5.5 min re-equilibration with the initial mobile phase conditions. Online mass spectrometry detection was performed using an Applied Biosystems/MDS Sciex API5000 Triple quadrupole mass spectrometer (ABsciex, Toronto, Canada), operating in positive electrospray ionization mode. The deoxyribonucleosides were monitored by multiple reaction monitoring using mass transitions 252.2→136.1 (dA), 228.2 → 112.1 (dC), 268.2→152.1 (dG), 243.2→127.0 (dT), 284.1→168.1 (8-oxodG), 244.1→128.0 (5-OHdC).

RNA sequencing. Liver and aortic tissues were homogenized by using a FastPrep®-24 instrument (MP Biomedical, Santa Ana, CA) and total RNA isolated using AllPrep DNA/RNA/Protein Mini Kit (Qiagen). mRNA levels from pooled animals (*Apoe*$^{-/-}$ [n=7] and *Apoe*$^{-/-}$*Neil3*$^{-/-}$ [n=9]) were compared using RNA sequencing analysis. The RNA sequencing and bioinformatic analyses were performed at BGI Tech Solutions Co., Ltd, Hong Kong. The sequencing was performed on an Illumina Hiseq 2000 machine, checking the quality of all samples with an Agilent 2100 Bioanalyzer. Low quality reads were removed according to BGI protocols. Genes were aligned to the mouse reference genome using Soap (2.21) and reads per kilobase of transcript per million mapped reads (RPKM) values were calculated[24].

Immunophenotyping of leukocytes. Immunophenotypic screening of blood, spleen, lymph nodes and thymus was performed by flow cytometry. Absolute leukocyte counts in blood were obtained using BD Trucount tubes, according to manufacturer’s protocol (BD). Briefly, Fc-receptor blocking antibody (anti Cd16/Cd32; eBioscience) was added to 50 µl anti-coagulated whole blood in Trucount tubes. After incubation at room temperature (10 minutes) the Trucount antibody cocktail (Supplemental Table 9) was added and the antibody/blood mix incubated for another 20 minutes at room temperature. Finally, samples were analyzed after incubation with hypotonic lysis buffer. Myeloid progenitor densities and mature leukocytes were measured in

spleen, and T-cell subsets were measured in blood, spleen, pooled lymph nodes, and thymus. Single cell suspensions of blood and spleen were prepared as described[25]. Lymph nodes and thymi were crushed over a 70 μm cell strainer (BD) and the strainers were flushed with ice-cold PBS. One femur and tibia per mouse were flushed with ice-cold PBS and single cell suspensions were prepared using a 70 μm cell strainer (BD). After blocking non-specific Fc-receptor binding (anti-Cd16/Cd32 antibody; eBioscience), complete cell pools were stained with the DIF or T-cell antibody mix (Supplemental Table 9) and incubated for 30 minutes. Cells were washed once and subsequently resuspended in 200 μl buffer (1xPBS, 5% BSA, 1 mM EDTA). All samples and buffers were kept on ice throughout the experiment unless indicated otherwise. All measurements were performed on a FACS Canto II (BD) and analysis of acquired data was performed using FACS Diva software (BD) and FlowJo 7.6.5 (Tree Star, Ashland, Oregon).

Measurement of body composition with DEXA Lunar PIXImus Densitometer. Body composition was measured at 26 weeks of age by dual-energy X-ray absorptiometry using a DEXA Lunar PIXImus Densitometer (GE Medical Systems, Fitchburg, WI). Prior to the x-ray analysis the mice were anaesthetized with i.p. injection of 0.1 ml per 20 g body weight of Hypnorm-Dormicum (1.25 mg/ml Dormicum, 2.5 mg/ml Fluanisone, and 0.079 mg/ml Fentanyl citrate). As the image area was not large enough for the whole mouse body, the analyses of lean body mass and fat mass were performed by using a defined region of interest (ROI), comprising from the start of the tail to the costophrenic angle (the angle between the diaphragm and the lung).

Genotyping of mice. Genotyping of *Apoe*$^{-/-}$ and *Apoe*$^{-/-}$*Neil3*$^{-/-}$ mice was carried out by use of the HotSHOT method for DNA preparation. DNA was mixed with specific primers (primer sequences can be provided upon request), PCR MasterMix (Promega, Madison, WI) and H_2O and amplified by using the GeneAmp 9700 PCR System (Applied Biosystems, Foster City, CA). The

amplification protocol was as follows: 95 °C; 3minutes, 35 cycles of [95°C; 30 seconds, 60°C (Neil3)/55 °C (Apoe); 30 sec, 72 °C; 30seconds], and final extension at 72°C; 3 minutes. The PCR products were visualized on a 1.5% agarose gel (Sigma-Aldrich).

RT-qPCR analysis. Total RNA was isolated from human atherosclerotic plaques, human PBMC, THP-1 cells and murine tissues with the use of RNeasy spin columns (Qiagen). The RNA was treated with DNase (Qiagen) and stored at -80°C until analysis. RNA concentrations and purity were assessed by spectrophotometer absorbance (NanoDrop ND-1000 Thermo Scientific, Wilmington, DE). Synthesis of cDNA was performed using q-Script cDNA Synthesis kit (Quanta Bioscience, Gaithersburg, MD). Quantification of mRNA was performed using Perfecta SYBR Green Fastmix ROX (Quanta Bioscience) and specific primer sets or TaqMan assays (Applied Biosystems, Foster City, CA) and the 7900HT Fast Real-Time PCR System (Applied Biosystems) with the accompanying software SDS 2.4. All primer sequences can be provided upon request. For each transcript, RT-qPCR was conducted in duplicates. Target transcript levels were quantified by the comparative Ct method using the reference gene *β-ACTIN* as endogenous control.

Statistical analysis. Except for the RNA sequencing analysis and experiments with a total sample size of ≤7, all data were analyzed using non-parametric tests, i.e., the Mann–Whitney U test, Spearman's rank correlation or Kruskal-Wallis test with Dunn's post test for multiple comparisons. Experiments with a total sample size of ≤ 7 were analyzed using two-tailed unpaired Student's *t* test and categorical data were analyzed using the chi-square test. Data are presented as median and interquartile range unless otherwise stated. Analyses were performed using Prism version 6.0 (GraphPad software, La Jolla, CA) and SPSS for Windows statistical software (version 18.0; SPSS Inc., Chicago, IL), and p values <0.05 were considered statistically significant.

In RNA sequencing analyses, differentially expressed genes (DEGs) were calculated based on RPKM values, assuming a Poisson distribution[26]. DEGs were defined as having a log2 ratio≥1 and a false discovery rate (FDR) <0.001. A KEGG pathway enrichment analysis of DEGs was performed using a hypergeometric distribution test model and pathways were ranked according to their level of significance. In figure 4, enrichment is reported as Q values, which is the FDR corrected p value of the enrichment analysis. A Q value <0.05 was considered as significant.

Gene list enrichment based on functional annotations and protein interactions network on differentially expressed gene signatures was done using the GATHER software package (http://gather.genome.duke.edu)[27] and Toppfun (ToppGene Suite; https://toppgene.cchmc.org)[28]. Transcription factor enrichment was based on experimentally proven binding sites and consensus binding sequences for eukaryotic transcription factors (positional weight matrices) and known transcription factor regulated genes (as listed in TRANSFAC v7.0; GATHER). All output was FDR corrected (P<0.05), while statistical significance was calculated based on the probability of seeing a Bayes factor or gene set enrichment of a particular magnitude in a given query.

REFERENCES

1 Perisic, L. *et al.* Gene expression signatures, pathways and networks in carotid atherosclerosis. *J. Int. Med.* **279,** 293-308 (2016).

2 Razuvaev, A. *et al.* Correlations between clinical variables and gene-expression profiles in carotid plaque instability. *Eur. J. Vasc. Endovasc. Surg.* **42,** 722-730 (2011).

3 Agardh, H. E. *et al.* Expression of fatty acid-binding protein 4/aP2 is correlated with plaque instability in carotid atherosclerosis. *J. Intern. Med.* **269,** 200-210 (2011).

4 Gregersen, I. *et al.* Increased systemic and local interleukin 9 levels in patients with carotid and coronary atherosclerosis. *PloS One.* **8,** e72769 (2013).

5 Abbas, A. *et al.* High levels of S100A12 are associated with recent plaque symptomatology in patients with carotid atherosclerosis. *Stroke.* **43,** 1347-1353 (2012).

6 Abbas, A. *et al.* Interleukin 23 levels are increased in carotid atherosclerosis: possible role for the interleukin 23/interleukin 17 axis. *Stroke.* **46,** 793-799 (2015).

7 Folkersen, L. *et al.* Prediction of ischemic events on the basis of transcriptomic and genomic profiling in patients undergoing carotid endarterectomy. *Mol. Med..* **18,** 669-675 (2012).

8 Irizarry, R. A. *et al.* Exploration, normalization, and summaries of high density oligonucleotide array probe level data. *Biostatistics.* **4,** 249-264 (2003).

9 Samstad, E. O. *et al.* Cholesterol crystals induce complement-dependent inflammasome activation and cytokine release. *J. Immunol.* **192,** 2837-2845 (2014).

10 Sejersted, Y. *et al.* Endonuclease VIII-like 3 (Neil3) DNA glycosylase promotes neurogenesis induced by hypoxia-ischemia. *Proc. Natl. Acad. Sci. USA.* **108,** 18802-18807 (2011).

11 Nicoletti, A., Kaveri, S., Caligiuri, G., Bariety, J. & Hansson, G. K. Immunoglobulin treatment reduces atherosclerosis in apo E knockout mice. *J. Clin. Invest.* **102,** 910-918 (1998).

12 Bligh, E. G. & Dyer, W. J. A rapid method of total lipid extraction and purification. *Can. J. Biochem. Physiol.* **37,** 911-917 (1959).

13 Oie, E. *et al.* Tetradecylthioacetic acid increases fat metabolism and improves cardiac function in experimental heart failure. *Lipids.* **48,** 139-154 (2013).

14 Bjorndal, B. *et al.* Krill powder increases liver lipid catabolism and reduces glucose mobilization in tumor necrosis factor-alpha transgenic mice fed a high-fat diet. *Metabolism.* **61,** 1461-1472 (2012).

15 Vernez, L., Wenk, M. & Krahenbuhl, S. Determination of carnitine and acylcarnitines in plasma by high-performance liquid chromatography/electrospray ionization ion trap tandem mass spectrometry. *Rapid Commun. Mass Spectrom.* **18,** 1233-1238 (2004).

16 Vigerust, N. F. *et al.* Free carnitine and acylcarnitines in obese patients with polycystic ovary syndrome and effects of pioglitazone treatment. *Fertil. Steril.* **98,** 1620-1626.e1621 (2012).

17 Bjorndal, B. *et al.* Dietary supplementation of herring roe and milt enhances hepatic fatty acid catabolism in female mice transgenic for hTNFalpha. *Eur. J. Nutr.* **51,** 741-753 (2012).

18 Bremer, J. The effect of fasting on the activity of liver carnitine palmitoyltransferase and its inhibition by malonyl-CoA. *Biochim. Biophys. Acta.* **665,** 628-631 (1981).

19 Vik, R. *et al.* Hypolipidemic effect of dietary water-soluble protein extract from chicken: impact on genes regulating hepatic lipid and bile acid metabolism. *Eur. J. Nutr.* (2014).

20 Skorve, J. *et al.* On the mechanism of the hypolipidemic effect of sulfur-substituted hexadecanedioic acid (3-thiadicarboxylic acid) in normolipidemic rats. *J. Lipid Res.* **34,** 1177-1185 (1993).

21 Hume, D. A. & Gordon, S. Optimal conditions for proliferation of bone marrow-derived mouse macrophages in culture: the roles of CSF-1, serum, Ca2+, and adherence. *J. Cell. Physiol.* **117,** 189-194 (1983).

22 Bryder, D. *et al.* Self-renewal of multipotent long-term repopulating hematopoietic stem cells is negatively regulated by Fas and tumor necrosis factor receptor activation. *J. Exp. Med.* **194,** 941-952 (2001).

23 Strand, J. M., Scheffler, K., Bjoras, M. & Eide, L. The distribution of DNA damage is defined by region-specific susceptibility to DNA damage formation rather than repair differences. *DNA Repair (Amst).* **18,** 44-51 (2014).

24 Mortazavi, A., Williams, B. A., McCue, K., Schaeffer, L. & Wold, B. Mapping and quantifying mammalian transcriptomes by RNA-Seq. *Nat Methods.* **5,** 621-628 (2008).

25 Otten, J. J. *et al.* Hematopoietic G-protein-coupled receptor kinase 2 deficiency decreases atherosclerotic lesion formation in LDL receptor-knockout mice. *FASEB J.* **27,** 265-276 (2013).

26 Audic, S. & Claverie, J. M. The significance of digital gene expression profiles. *Genome Res.* **7,** 986-995 (1997).

27 Chang, J. T. & Nevins, J. R. GATHER: a systems approach to interpreting genomic signatures. *Bioinformatics.* **22,** 2926-2933 (2006).

28 Chen, J., Bardes, E. E., Aronow, B. J. & Jegga, A. G. ToppGene Suite for gene list enrichment analysis and candidate gene prioritization. *Nucleic Acids Res.* **37,** W305-311 (2009).

Table S1. Clinical characterization of patients with carotid atherosclerosis (*Biobank of Karolinska Endarterectomies (BIKE)*)

	**Symptomatic N=71**	**Asymptomatic N=35**	**p**
**Age^{A}, years**	78 (66-78)	69 (60-73)	0.0067
**Male sex, % (n)**	71.8 (51)	97.1 (34)	0.0049
**BMI ^{A}, kg/m^{2}**	25 (24-28)	26 (25-28)	0.069
**Current smoking, % (n)**	59.7(37)	53.1 (17)	0.7
**Hypertension, % (n)**	87.3 (62)	85.7 (30)	1
**Diabetes mellitus, % (n)**	25.4 (18)	22.9 (8)	0.97
**ASA treatment, % (n)**	84.5 (60)	85.7 (30)	1
**Statin treatment, % (n)**	91.5 (65)	94.3 (33)	0.91
**CRP^{A}, mg/l**	2.8 (1.3-7.1)	3.2 (0.86-4.4)	0.17
**Total cholesterol ^{A}, mM**	4.2 (3.8-5.4)	4.3 (3.6-5.2)	0.98
**LDL cholesterol^{A}, mM**	2.3 (1.9-3.2)	2.3 (1.9-3)	0.79
**HDL cholesterol^{A}, mM**	1.2 (0.92-1.4)	1.1 (0.9-1.3)	0.3
**Triglycerides^{A}, mM**	1.4 (0.93-2.1)	1.6 (1.2-2.2)	0.27
**HbA1c^{A}, %**	5 (4.6-6.1)	4.9 (4.7-5.5)	0.99

BMI, body mass index; ASA, acetylsalicylic acid; Symptomatic, relevant symptoms within the last 6 months before surgery; Asymptomatic, no symptoms or no relevant symptoms within the last 6 months before surgery. Clinical symptoms included stroke, TIA and amaurosis fugax ipsilateral to the stenotic carotid artery. Data were analyzed using Mann-Whitney U test or chi-square test and are given as percentage (numbers) or $^{(A)}$median (interquartile range).

Table S2. Clinical characterization of patients with carotid atherosclerosis (Biobank of Oslo)

	Symptomatic N=54	Asymptomatic N=14	p
**Age^{A}, years**	68.5 (52-81)	69.5 (48-79)	0.98
**Male sex, % (n)**	69 (37)	86 (12)	0.21
**BMI ^{A}, kg/m^{2}**	25.0 (18.4-33.0)	25.1 (20.0-32.0)	0.47
**Current smoking, % (n)**	50 (27)	50 (7)	1.00
**Hypertension, % (n)**	72 (39)	79 (11)	0.64
**Diabetes mellitus, % (n)**	22 (12)	36 (5)	0.31
**ASA treatment, % (n)**	93 (50)	86 (12)	0.43
**Statin treatment, % (n)**	93 (50)	93 (13)	0.97
**CRP^{A}, mg/l**	3.5 (1.0-39.0)	4.5 (1.0-55.0)	0.62
**Leukocyte count ^{A}, 10^{9}/l**	8.3 (2.4-12.0)	6.7 (3.8-10.2)	0.02
**Platelets^{A}, 10^{9}/l**	292 (141-493)	285 (180-411)	0.99
**Total cholesterol ^{A}, mM**	4.1 (2.7-7.5)	4.2 (3.2-4.8)	0.96
**LDL cholesterol^{A}, mM**	2.4 (1.2-4.5)	2.6 (2.0-2.8)	0.52
**HDL cholesterol^{A}, mM**	1.3 (0.8-2.7)	1.3 (0.8-1.8)	0.42
**Triglycerides^{A}, mM**	1.3 (0.5-3.8)	1.4 (0.5-2.5)	0.90
**HbA1c^{A}, %**	5.8 (4.4-9.5)	6.4 (5.0-12.5)	0.23

BMI, body mass index; ASA, acetylsalicylic acid; Symptomatic, relevant symptoms within the last 6 months before surgery; Asymptomatic, no symptoms or no relevant symptoms within the last 6 months before surgery. Clinical symptoms included stroke, TIA and amaurosis fugax ipsilateral to the stenotic carotid artery. Data were analyzed using Mann-Whitney U test or chi-square test and are given as percentage (numbers) or $^{(A)}$median (interquartile range).

Table S3. Plasma levels of cytokines in *Apoe*$^{-/-}$ and *Apoe*$^{-/-}$*Neil3*$^{-/-}$ mice

Cytokine (pg/ml)	*Apoe*$^{-/-}$	*Apoe*$^{-/-}$*Neil3*$^{-/-}$	p values
**Il-1α**	41 (31-55)	44 (30-49)	0.64
**Il-1β**	740 (583-1064)	440 (242-690)	0.014
**Il-2**	33 (29-87)	43 (22-123)	0.50
**Il-3**	48 (18-95)	24 (15-27)	0.40
**Il-5**	78 (51-95)	40 (25-67)	0.009
**Il-6**	37 (28-48)	24 (20-35)	0.06
**Il-10**	277 (210-305)	213 (160-323)	0.49
**Il-12p40**	401 (366-458)	381 (328-419)	0.39
**Il-12**	436 (297-523)	431 (170-557)	0.91
**Il-13**	794 (398-1139)	476 (286-996)	0.41
**Il-17**	116 (81-247)	130 (77-364)	0.76
**Ccl11**	2203 (1711-2918)	2048 (1700-2583)	0.69
**G-CSF**	212 (153-298)	139 (79-203)	0.025
**GM-CSF**	665 (556-731)	561 (461-734)	0.39
**Ifn-γ**	101 (73-152)	106 (77-127)	0.86
**Ccl2**	743 (570-1161)	643 (489-755)	0.19
**Ccl3**	80 (69-88)	64 (50-88)	0.14
**Ccl4**	136 (104-266)	111 (98-151)	0.32
**Ccl5**	29 (22-44)	29 (18-38)	0.59
**Kc**	135 (106-417)	104 (86-138)	0.22
**Tnf**	735 (521-1255)	494 (373-791)	0.099

G-CSF, granulocyte colony stimulating factor; GM-CSF, granulocyte-macrophage CSF; Ifn, interferon; Kc, keratinocyte chemoattractant; Tnf, tumor necrosis factor. Data are presented as median and interquartile range and were analyzed using the Mann-Whitney U test (n=7-12).

Table S4. Metabolic parameters in *Apoe*$^{-/-}$ and *Apoe*$^{-/-}$*Neil3*$^{-/-}$ mice

	***Apoe*$^{-/-}$**	***Apoe*$^{-/-}$*Neil3*$^{-/-}$**	**p values**
**ROI Fat^{A}, % of body weight**	37 (32-41)	34 (30-36)	0.30
**ROI Lean^{A}, % of body weight**	63 (59-68)	66 (64-70)	0.30
**Body weight^{B}, gram**	36.6 (34.9-40.1)	36.5 (33.2-40.0)	0.40
**Food intake^{C}, gram/gram body weight/24 hours**	0.12 (0.05-0.19)	0.19 (0.09-0.24)	0.18
**Non-fasting glucose^{C}, mmol/l**	9.6 (9.0-10.3)	10.3 (8.3-10.8)	0.50
**Phospholipids^{C}, mmol/l**	3.5 (2.8-4.7)	4.7 (4.1-5.2)	0.20

ROI (region of interest), comprising from the start of the tail to the costophrenic angle, as evaluated by dual-energy X-ray absorptiometry. Data are presented as median and interquartile range and were analyzed using the Mann-Whitney U test (^{A}n=8-12, ^{B}n=28-30, ^{C}n=6-8).

Table S5. Lipid and FA levels in the livers of *Apoe*$^{-/-}$ and *Apoe*$^{-/-}$*Neil3*$^{-/-}$ mice

Liver, non-fasting values	*Apoe*$^{-/-}$	*Apoe*$^{-/-}$*Neil3*$^{-/-}$	p values
**Cholesterol, µmol/g liver**	25.9 (21.4-29.4)	31.7 (23.9-34.8)	0.15
**Phospholipids, µmol/g liver**	17.7 (16.7-20.1)	19.1 (16.9-19.9)	0.90
**MUFA, Wt%**	62.5 (59.3-65.1)	66.5 (65.7-68.4)	0.002
**C18:1n-9 , Wt%**	51.3 (49.4-54.9)	55.7 (54.7-57.8)	0.007
**C18:1n-7, Wt%**	3.34 (3.11-3.87)	4.13 (3.77-4.27)	0.03
**PUFA, Wt%**	13.34 (9.53-15.31)	8.86 (8.00-11.09)	0.04
**n-9 PUFA, Wt%**	1.79 (1.04-1.99)	0.86 (0.77-1.19)	0.02
**n-6 PUFA, Wt%**	9.70 (7.20-11.50)	6.80 (6.10-8.40)	0.04
**n-3 PUFA, Wt%**	1.60 (1.30-2.10)	1.37 (1.03-1.66)	0.18
**C20:4n-6 arachidonic acid, Wt%**	4.68 (3.10-5.54)	3.00 (2.32-3.76)	0.04
**C20:5n-3 eicosapentaenoic acid, Wt%**	0.08 (0.07-0.09)	0.06 (0.05-0.08)	0.03

FA, fatty acids; MUFA, monounsaturated fatty acids; PUFA, polyunsaturated fatty acids; Wt%, weight percent. Data are presented as median and interquartile range and were analyzed using the Mann-Whitney U test (n=7-9).

Table S6. Plasma levels of carnitine derivatives and FA in *Apoe*$^{-/-}$ and *Apoe*$^{-/-}$*Neil3*$^{-/-}$ mice.

**Plasma, non-fasting**	***Apoe*$^{-/-}$**	***Apoe*$^{-/-}$*Neil3*$^{-/-}$**	**p values**
**Acetyl Carnitine, µM**	6.20 (6.00-8.10)	7.34 (6.70-9.70)	0.054
**Palmitoyl Carnitine, µM**	0.24 (0.22-0.33)	0.42 (0.36-0.49)	0.004
**Free FA, µM**	0.25 (0.10-0.32)	0.29 (0.21-0.39)	0.30
**Absolute levels of FA, µg FA/ml**	4602 (3614-6838)	7117 (5332-8529)	0.083

Data are presented as median and interquartile range and were analyzed using the Mann-Whitney U test (n=7-12).

Table S7. Gene enrichment analysis of differentially expressed genes (DEGs) in thoracic aortas of *Apoe*$^{-/-}$*Neil3*$^{-/-}$ and *Apoe*$^{-/-}$ mice as assessed by RNA sequencing

#	ID	Description	DEGs	FDR-corrected p value
	**Mouse phenotype**			
1	MP:0000187	Abnormal triglyceride level	56	2.260e-8
2	MP:0002118	Abnormal lipid homeostasis	97	5.966e-8
3	MP:0001547	Abnormal lipid level	92	8.097e-8
4	MP:0003949	Abnormal circulating lipid level	77	1.530e-7
5	MP:0005318	Decreased triglyceride level	37	1.004e-6
	**Immunological genome project**			
6	GSM605865500	Myeloid Cells, MF.Thio5.II-480int.PC, Cd115+ MHCII- F480int SiglecF- Cd11c+, Peritoneal Cavity, avg-3	91	3.083e-38
7	GSM605862500	Myeloid Cells, MF.Thio5.II-480hi.PC, Cd115+ MHC II- F480hi SiglecF-, Peritoneal Cavity, avg-3	88	1.299e-35
8	GSM605856500	Myeloid Cells, MF.Thio5.II+480int.PC, Cd115+ MHC II+ F480lo SiglecF- Cd11c+, Peritoneal Cavity, avg-3	82	2.073e-31
	**Molecular Signature**			
9	M1920	Genes forming the macrophage-enriched metabolic network	159	3.034e-47

Table S8. Flow cytometry analyses of leukocyte subpopulations in spleen, blood, lymph nodes and thymus.

			Apoe$^{-/-}$	*Apoe*$^{-/-}$ *Neil3*$^{-/-}$	P values
**Spleen**		% B cells	47 (2)	53 (7)	0.44
		% T cells	23 (3)	24 (1)	0.86
		% Granulocytes	6.9 (2.8)	2.8 (1.9)	0.29
		% Monocytes	6.2 (1.5)	4.0 (1.6)	0.38
		% NK cells	2.5 (0.3)	3.0 (0.6)	0.47
	Monocytes	% Ly6C+	33 (3)	32 (4)	0.82
		% Ly6C-	67 (3)	68 (4)	0.82
	Cd3+ T cells	% Cd4+	56 (1)	56 (1)	0.66
		% Cd8+	35.0 (0.3)	37 (1)	0.20
**Blood**		B cells[A]	309 (150)	646 (426)	0.50
		T cells[A]	183 (60)	236 (96)	0.66
		Granulocytes[A]	726 (298)	535 (88)	0.57
		Monocytes[A]	212 (110)	150 (8)	0.60
		NK cells[A]	167 (94)	76 (5)	0.39
	Cd3+ T cells	% Cd4+	46 (3)	48 (1)	0.27
		% Cd8+	43 (1)	39 (3)	0.23
**Lymph nodes**		% Cd3+ T cells	36 (4)	40 (2)	0.49
		% Cd4+ T cells	19 (2)	19 (1)	0.95
		% Cd8+ T cells	13 (2)	17 (1)	0.25
**Thymus**		% Cd3+ T cells	13.0 (0.3)	13.0 (0.2)	0.95
		% Cd4+ T cells	6.6 (0.3)	6.6 (0.1)	0.98
		% Cd8+ T cells	2.4 (0.2)	2.4 (0.1)	0.71

Values represent % of cells or [(A)]number of cells/μl blood. Data are presented as mean (SEM) and were analyzed using Student's *t* test (n=3 in each group).

Table S9. Antibodies used for immunophenotypic screening of thymus, lymph nodes, spleen

Antibody	Conjugate	Company	Staining
**Cd3**	eFluor450	eBioscience	Trucount
**Cd3**	FITC	eBioscience	T-cell, DIF
**Cd4**	PerCP	BD	T-cell
**Cd4**	APC-H7	BD	Trucount, DIF
**Cd8α**	FITC	eBioscience	Trucount
**Cd8α**	eFluor450	eBioscience	T-cell, DIF
**Cd11b**	PE-Cy7	BD	Trucount, DIF
**Cd25**	APC	BD	T-cell
**Cd44**	APC-Cy7	BD	T-cell
**Cd45**	PerCP	Biolegend	Trucount
**Cd45RA (B220)**	V500	BD	Trucount, DIF
**Cd62L**	PE-Cy7	BD	T-cell
**Ly6C**	APC	Miltenyi	Trucount, DIF
**Ly6G**	PE	BD	DIF
**Ly6G**	APC-Cy7	BD	Trucount
**NK1.1**	PE	BD	Trucount
**NK1.1**	PerCP-Cy5.5	BD	DIF

Supplementary Figures with Legends

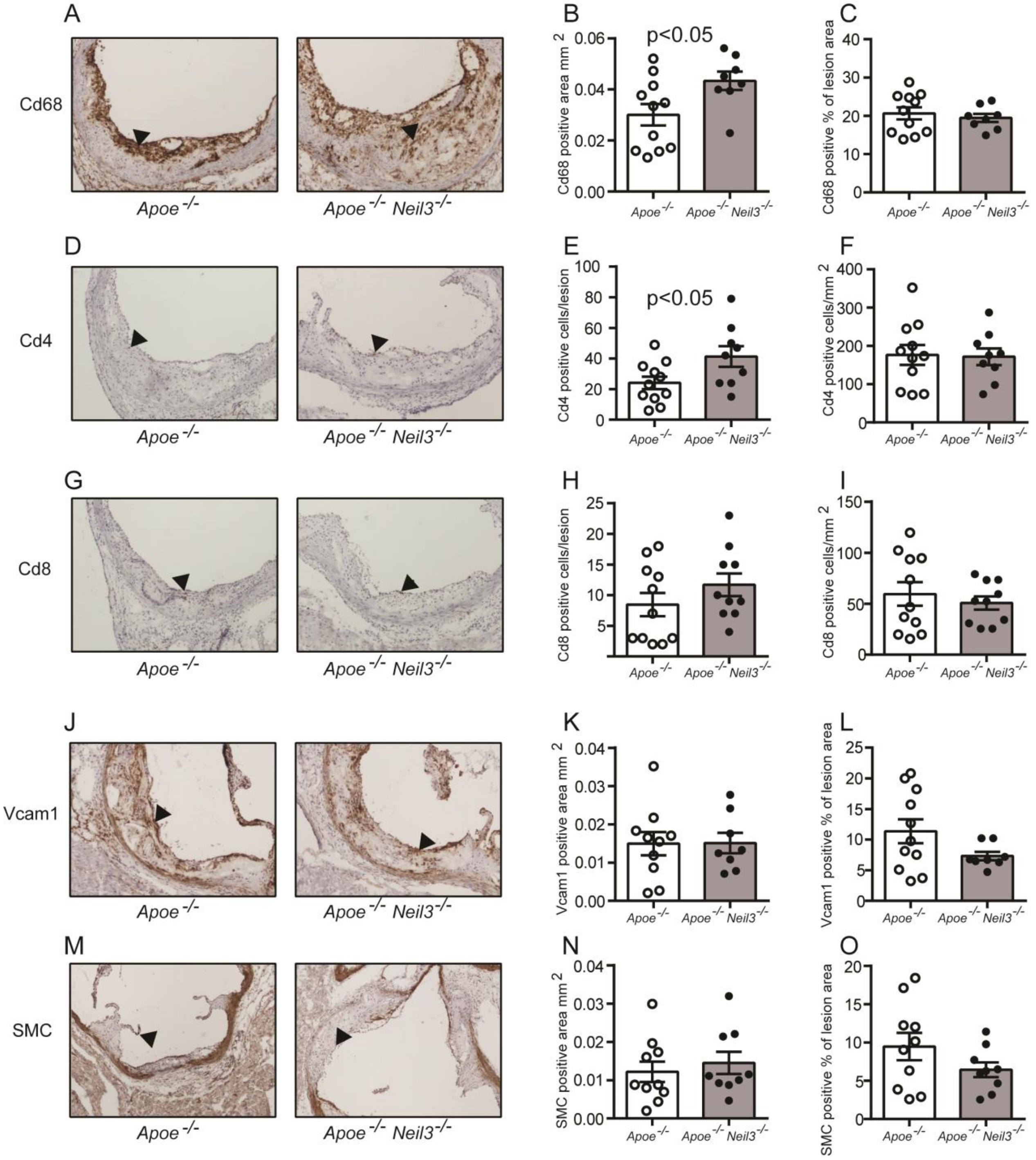

Figure S1. Immunohistochemical staining of the aortic sinus of *Apoe*$^{-/-}$ and *Apoe*$^{-/-}$*Neil3*$^{-/-}$ mice. Immunohistochemistry for Cd68 (**A**), Cd4 (**D**), Cd8 (**G**), Vcam1 (**J**) and smooth muscle

cell (SMC) α-actin (**M**). **B**) Absolute positive area (mm^2) stained for Cd68. **C**) Relative area stained positive for Cd68 (% of total lesion area). **E**) Number of positive cells stained for Cd4. **F**) Relative amount of $Cd4^+$ cells (positive cells/mm^2). **H**) Number of positive cells stained for Cd8. **I**) Relative amount of $Cd8^+$ cells (positive cells/mm^2). **K**) Absolute positive area (mm^2) stained for Vcam1. **L**) Relative area stained positive for Vcam1 (% of total lesion area). **N**) Absolute positive area (mm^2) stained for SMC α-actin. **O**) Relative area stained positive for SMC α-actin (% of total lesion area). Data are presented as single values, median and interquartile range and were analyzed using Mann-Whitney U test (n=8-11).

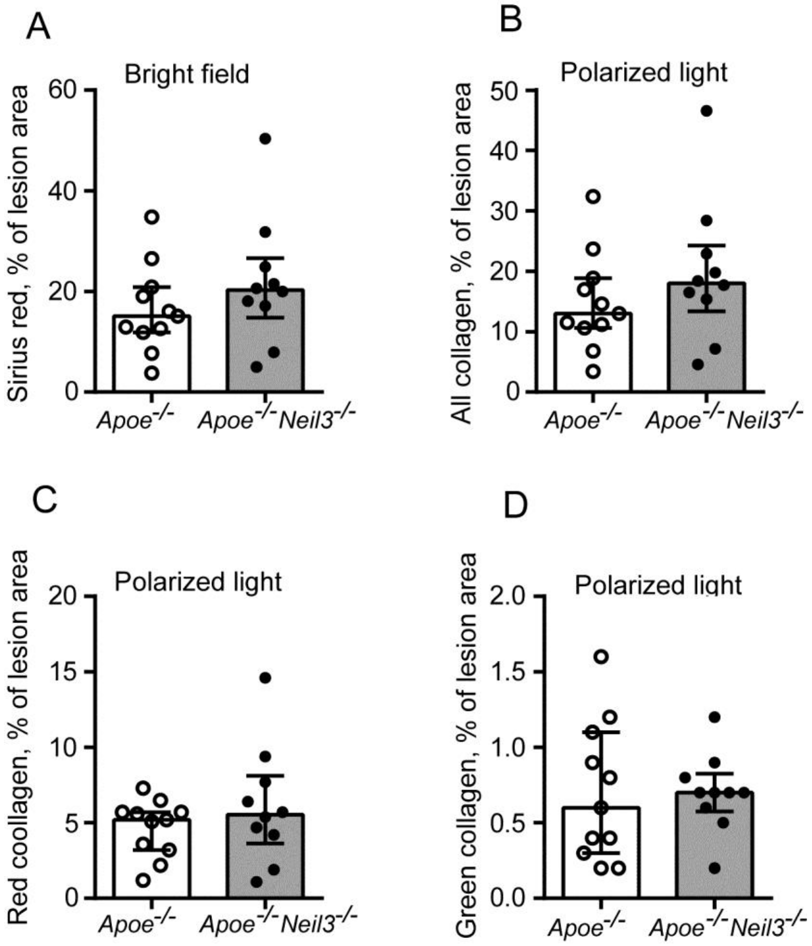

Figure S2. Quantification of collagen in the atherosclerotic lesions of the aortic root in *Apoe*$^{-/-}$ and *Apoe*$^{-/-}$*Neil3*$^{-/-}$ mice. Relative area stained positive for Sirius Red as evaluated by bright field microscopy, expressed as % lesion area (**A**). All collagen (**B**), red collagen (**C**) and green collagen (**D**), as evaluated under polarized light, expressed as % lesion area. Data are presented as single values, median and interquartile range and were analyzed using Mann-Whitney U test (n=10-11).

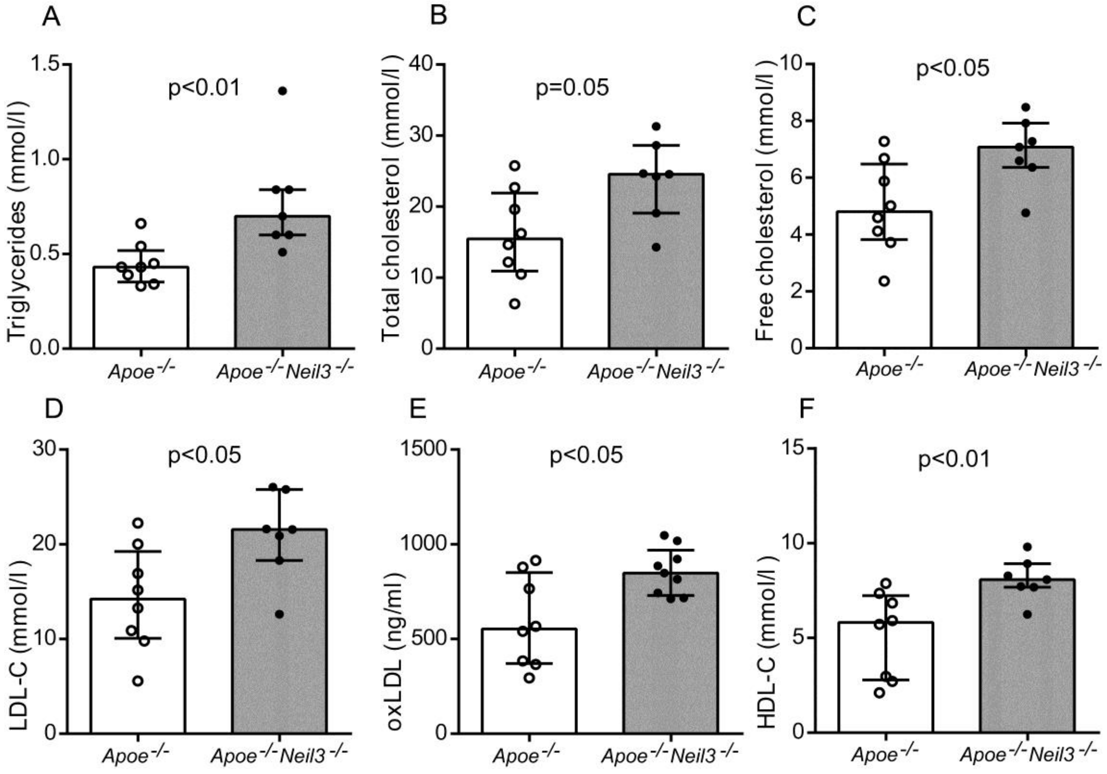

Figure S3. An atherogenic lipid profile in *Apoe$^{-/-}$Neil3$^{-/-}$* mice. A) TG, **B**) total cholesterol, **C**) free cholesterol, **D**) LDL cholesterol, **E**) oxLDL and **F**) HDL cholesterol; n=8-11(*Apoe$^{-/-}$*) and n=7-10 (*Apoe$^{-/-}$Neil3$^{-/-}$*). Data are non-fasting and presented as single values, median and interquartile range.

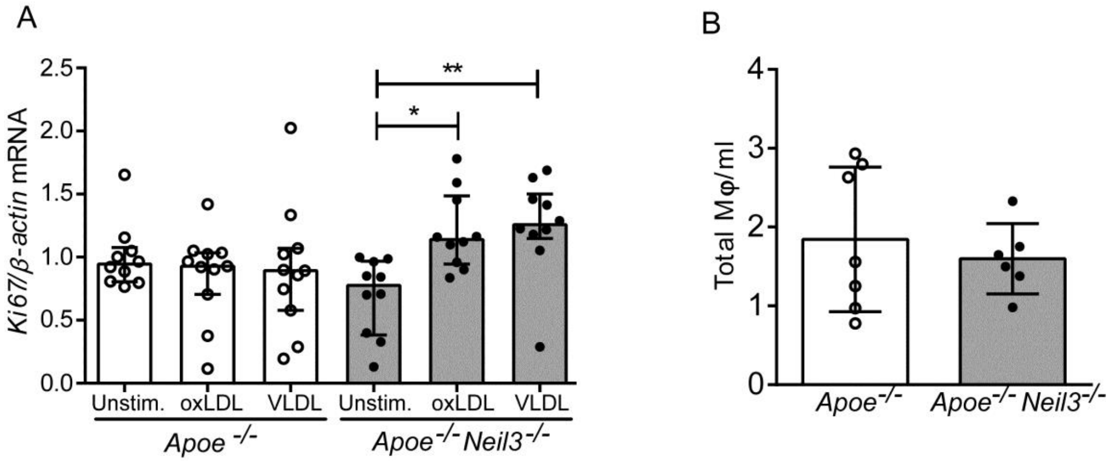

Figure S4. Increased proliferation in lipid-exposed bone marrow-derived macrophages (BMDM) from *Apoe*$^{-/-}$*Neil3*$^{-/-}$ mice. A) Pooled data from two separate experiments showing mRNA levels of the proliferation marker *Ki67*, evaluated by RT-qPCR in BMDM stimulated with oxLDL (20 µg/ml) and VLDL (25 µg/ml) for 3 hours. *Ki67* mRNA expression was normalized to *β–actin* as reference gene. All values are normalized to unstimulated BMDM from *Apoe*$^{-/-}$ mice. Data are presented by single values, median and interquartile range (n=10-12) and were analyzed using *Kruskall-Wallis test followed by Dunn's test and $^{\#}$Mann-Whitney U test. *p<0.05 and **p<0.001 versus unstimulated cells; #p<0.05 unstimulated versus unstimulated. **B**) Number of macrophages recruited to the peritoneal cavity as a response to sterile inflammation after peritoneal injection of 3% Brewer's thioglycollate (n=6-7). Data are presented as single values, median and interquartile range and were analyzed using Mann-Whitney U test.

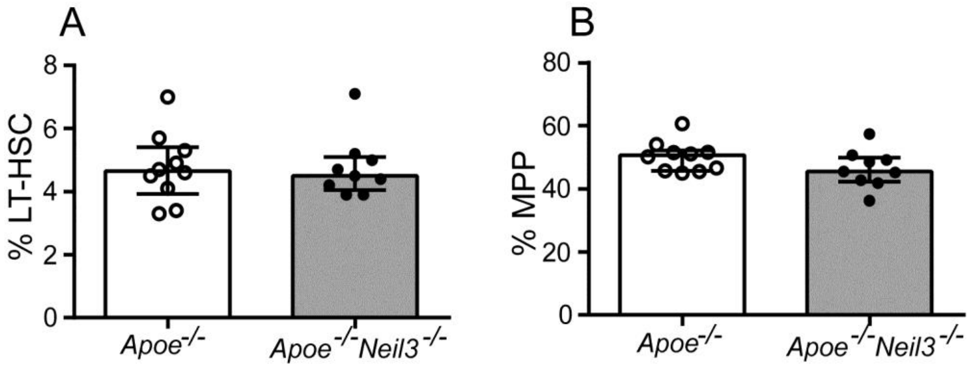

Figure S5. Flow cytometry analyses of bone marrow aspirates from *Apoe*$^{-/-}$ and *Apoe*$^{-/-}$*Neil3*$^{-/-}$ mice. A) Long-term hematopoietic stem cells (LT-HSC). **B**) Multipotent progenitor (MPP) cells. Data are presented as single values, median and interquartile range and were analyzed using Mann-Whitney U test (n=9-10).

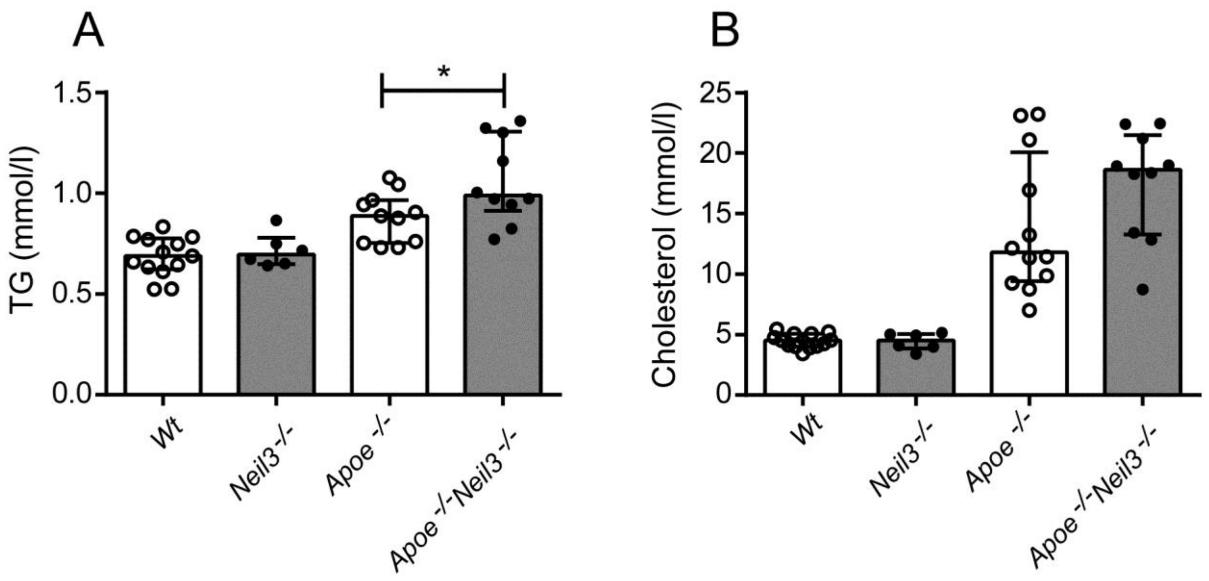

Figure S6. Serum Triglyceride- and Cholesterol levels in wild type (Wt), *Neil3*$^{-/-}$, *Apoe*$^{-/-}$ and *Apoe*$^{-/-}$*Neil3*$^{-/-}$ mice A) TG, **B**) total cholesterol, in 22 weeks old mice fed high-fat diet for 14 weeks. Blood samples were taken after 4 hours of fasting. Data are presented as single values, median and interquartile range (Wt; n=13, *Neil3*$^{-/-}$; n=6, *Apoe*$^{-/-}$; n=11, *Apoe*$^{-/-}$*Neil3*$^{-/-}$; n=10).
